# Parasitoid Serpins Evolve Novel Functions to Manipulate Host Homeostasis

**DOI:** 10.1093/molbev/msad269

**Published:** 2023-12-07

**Authors:** Zhiwei Wu, Ruizhong Yuan, Qijuan Gu, Xiaotong Wu, Licheng Gu, Xiqian Ye, Yuenan Zhou, Jianhua Huang, Zhizhi Wang, Xuexin Chen

**Affiliations:** Institute of Insect Sciences, College of Agriculture and Biotechnology, Zhejiang University, Hangzhou, China; Guangdong Lab for Lingnan Modern Agriculture, Guangzhou, China; Ministry of Agriculture Key Lab of Molecular Biology of Crop Pathogens and Insect Pests, Zhejiang University, Hangzhou, China; Key Laboratory of Biology of Crop Pathogens and Insects of Zhejiang Province, Zhejiang University, Hangzhou, China; Institute of Insect Sciences, College of Agriculture and Biotechnology, Zhejiang University, Hangzhou, China; Ministry of Agriculture Key Lab of Molecular Biology of Crop Pathogens and Insect Pests, Zhejiang University, Hangzhou, China; Key Laboratory of Biology of Crop Pathogens and Insects of Zhejiang Province, Zhejiang University, Hangzhou, China; Institute of Insect Sciences, College of Agriculture and Biotechnology, Zhejiang University, Hangzhou, China; Ministry of Agriculture Key Lab of Molecular Biology of Crop Pathogens and Insect Pests, Zhejiang University, Hangzhou, China; Key Laboratory of Biology of Crop Pathogens and Insects of Zhejiang Province, Zhejiang University, Hangzhou, China; Institute of Insect Sciences, College of Agriculture and Biotechnology, Zhejiang University, Hangzhou, China; Guangdong Lab for Lingnan Modern Agriculture, Guangzhou, China; Ministry of Agriculture Key Lab of Molecular Biology of Crop Pathogens and Insect Pests, Zhejiang University, Hangzhou, China; Key Laboratory of Biology of Crop Pathogens and Insects of Zhejiang Province, Zhejiang University, Hangzhou, China; Institute of Insect Sciences, College of Agriculture and Biotechnology, Zhejiang University, Hangzhou, China; Ministry of Agriculture Key Lab of Molecular Biology of Crop Pathogens and Insect Pests, Zhejiang University, Hangzhou, China; Key Laboratory of Biology of Crop Pathogens and Insects of Zhejiang Province, Zhejiang University, Hangzhou, China; Institute of Insect Sciences, College of Agriculture and Biotechnology, Zhejiang University, Hangzhou, China; Guangdong Lab for Lingnan Modern Agriculture, Guangzhou, China; Ministry of Agriculture Key Lab of Molecular Biology of Crop Pathogens and Insect Pests, Zhejiang University, Hangzhou, China; Key Laboratory of Biology of Crop Pathogens and Insects of Zhejiang Province, Zhejiang University, Hangzhou, China; Institute of Insect Sciences, College of Agriculture and Biotechnology, Zhejiang University, Hangzhou, China; Ministry of Agriculture Key Lab of Molecular Biology of Crop Pathogens and Insect Pests, Zhejiang University, Hangzhou, China; Key Laboratory of Biology of Crop Pathogens and Insects of Zhejiang Province, Zhejiang University, Hangzhou, China; Institute of Insect Sciences, College of Agriculture and Biotechnology, Zhejiang University, Hangzhou, China; Guangdong Lab for Lingnan Modern Agriculture, Guangzhou, China; Ministry of Agriculture Key Lab of Molecular Biology of Crop Pathogens and Insect Pests, Zhejiang University, Hangzhou, China; Key Laboratory of Biology of Crop Pathogens and Insects of Zhejiang Province, Zhejiang University, Hangzhou, China; State Key Lab of Rice Biology, Zhejiang University, Hangzhou, China; Institute of Insect Sciences, College of Agriculture and Biotechnology, Zhejiang University, Hangzhou, China; Guangdong Lab for Lingnan Modern Agriculture, Guangzhou, China; Ministry of Agriculture Key Lab of Molecular Biology of Crop Pathogens and Insect Pests, Zhejiang University, Hangzhou, China; Key Laboratory of Biology of Crop Pathogens and Insects of Zhejiang Province, Zhejiang University, Hangzhou, China; State Key Lab of Rice Biology, Zhejiang University, Hangzhou, China; The Rural Development Academy, Zhejiang University, Hangzhou, China; Institute of Insect Sciences, College of Agriculture and Biotechnology, Zhejiang University, Hangzhou, China; Guangdong Lab for Lingnan Modern Agriculture, Guangzhou, China; Ministry of Agriculture Key Lab of Molecular Biology of Crop Pathogens and Insect Pests, Zhejiang University, Hangzhou, China; Key Laboratory of Biology of Crop Pathogens and Insects of Zhejiang Province, Zhejiang University, Hangzhou, China; State Key Lab of Rice Biology, Zhejiang University, Hangzhou, China

**Keywords:** parasitoid wasp, teratocytes, serpin, adaptive evolution, antimicrobial activity, carbohydrate metabolism

## Abstract

Parasitoids introduce various virulence factors when parasitism occurs, and some taxa generate teratocytes to manipulate the host immune system and metabolic homeostasis for the survival and development of their progeny. Host-parasitoid interactions are extremely diverse and complex, yet the evolutionary dynamics are still poorly understood. A category of serpin genes, named *CvT-serpin*s, was discovered to be specifically expressed and secreted by the teratocytes of *Cotesia vestalis*, an endoparasitoid of the diamondback moth *Plutella xylostella*. Genomic and phylogenetic analysis indicated that the *C. vestalis* serpin genes are duplicated and most of them are clustered into 1 monophyletic clade. Intense positive selection was detected at the residues around the P1–P1′ cleavage sites of the Cv-serpin reactive center loop domain. Functional analyses revealed that, in addition to the conserved function of melanization inhibition (CvT-serpins 1, 16, 18, and 21), CvT-serpins exhibited novel functions, i.e. bacteriostasis (CvT-serpins 3 and 5) and nutrient metabolism regulation (CvT-serpins 8 and 10). When the host-parasitoid system is challenged with foreign bacteria, CvT-serpins act as an immune regulator to reprogram the host immune system through sustained inhibition of host melanization while simultaneously functioning as immune effectors to compensate for this suppression. In addition, we provided evidence that CvT-serpin8 and 10 participate in the regulation of host trehalose and lipid levels by affecting genes involved in these metabolic pathways. These findings illustrate an exquisite tactic by which parasitoids win out in the parasite–host evolutionary arms race by manipulating host immune and nutrition homeostasis via adaptive gene evolution and neofunctionalization.

## Introduction

Parasitism is a widespread type of symbiosis in nature, and increasing focus has been dedicated to the interactions between parasites and their hosts (Poulin and Morand 2000). Parasites are a class of organisms that live in or attach to another organism (host) to obtain the nutrition or shelter required for their survival, development, or reproduction at the cost of utilizing and consuming the host (Poulin and Morand 2000; Poulin 2011). The parasite–host arms race is exceedingly complex, reflecting long periods of coevolution (Ameline et al. 2021; Gasmi et al. 2021; Hu et al. 2021; Huang et al. 2021). Nevertheless, much of the genomic signature remains unknown.

Parasitic wasps, also known as hymenopteran parasitoids, are among the most diverse animals in the world. Adult females lay eggs on or inside the host, and the development of hatched offspring ultimately leads to the death of the host, making them excellent agents for pest control (Wang et al. 2018). Parasitic wasps have evolved various strategies to ensure successful parasitism, including the introduction of virulence factors, such as venom and polydnaviruses (PDVs), into the host hemocoel to modulate the host's immune response and metabolic homeostasis (Huang et al. 2021; Wang et al. 2021a; Gao et al. 2022; Wu et al. 2022; Zhou et al. 2022). In some parasitic wasps, when endoparasitoid eggs hatch, giant cells called teratocytes are dissociated from the embryonic cellular membrane and released into the hemocoel of the host, which is essential for host homeostasis regulation to provide an advantageous environment for the development of their offspring (Nakamatsu et al. 2001; Nakamatsu et al. 2002; Nakamatsu and Tanaka 2003; Strand 2014; Moreau and Asgari 2015; Strand and Burke 2020). Although the fundamental physiological functions of teratocytes have long been recognized, little is known about their essential components and regulatory mechanisms. From an evolutionary perspective, while some PDV and venom proteins exhibit signs of parasitoid–host coevolution, the evolutionary significance of the proteins expressed in teratocytes has largely been underestimated (Herniou et al. 2004; Jancek et al. 2013; Martinson et al. 2016; Burke et al. 2018).

Serine protease inhibitors (serpins), which occur in all kingdoms of life, are one of the largest protease inhibitor superfamilies in nature (Law et al. 2006; Spence et al. 2021). Most serpins share a conserved tertiary structure with an exposed reactive center loop (RCL) that contains a cleavage site between residues P1 and P1′ and residues were incrementing numbering along the N-terminal direction of the cleaved peptide bond (P2, P3, P4, and etc.) and in the same way on the carboxyl side of the cleavage site (P1′, P2′, P3′, and etc.) (Meekins et al. 2017). Once a serpin binds the target protein, its cleavage site is cleaved, followed by the formation of a covalent complex and consequent inactivation of the target protein (Gettins 2002; Whisstock and Bottomley 2006; Huntington 2011). Vertebrate serpins are well studied, especially in *Homo sapiens*, and their extensive functions are involved in the inflammatory response, the immune response, tumor formation and metastasis, blood agglutination, senility, sperm development, and the regulation of glucose and lipid metabolism (El Ouaamari et al. 2016; Yang and Geiger 2017; Choi et al. 2019). For instance, a plasma serpin, *Homo sapiens* serpinA1, has been found to bind with proteins such as apolipoprotein and glucose-regulated proteins to maintain metabolic homeostasis, and a deficiency in serpinA1 can result in a series of metabolic diseases such as atheroma and diabetes mellitus (Mashiba et al. 2001; Pagetta et al. 2003; Finotti and Pagetta 2004; O’Brien et al. 2020, 2022; Rabekova et al. 2021). Moreover, *Homo sapiens* serpinA12 has been proven to regulate blood glucose metabolism levels by inhibiting human kallikrein 7, which cleaves human insulin, via a classical serpin mechanism (Heiker et al. 2013; Salek Maghsoudi et al. 2022). Gene duplication is an important process in the origin of new traits, providing the original genetic material for evolution and various biological functions of offspring (Pottier et al. 2022). The functional diversity of the serpin superfamily may result from a battery of gene duplication events (Askew et al. 2004). It is well illustrated by the evidence that evolutionary duplicated *Mus musculus* serpin*B3a-d* share high sequence identity but target distinct proteases and perform different inhibitory functions, *Homo sapiens* serpinB3-B4 arising from tandem gene duplication also exhibited the same phenomenon (Al-Khunaizi et al. 2002; Askew et al. 2004; Sun et al. 2017). In addition, a series of serpin gene duplication events that could lead to new functions emerged in the genomes of *Gallus gallus* and *Brachydanio rerio* (Benarafa and Remold-O’Donnell 2005). In contrast to vertebrate serpins, the majority of studies on insect serpin functions are limited to immune modulation. Most insect serpins have been found to regulate the immune response, including but not limited to serpins from *Drosophila melanogaster* (Reichhart et al. 2011; Katsukawa et al. 2018), *Manduca sexta* (Tong et al. 2005; Zou and Jiang 2005; An et al. 2011; Suwanchaichinda et al. 2013; He et al. 2017; Wang et al. 2017; Wang et al. 2020a), *Bombyx mori* (Liu et al. 2015a; Li et al. 2016a; Li et al. 2017; Wang et al. 2019), and *Tenebrio molitor* (Jiang et al. 2009). Like most insect serpins, parasitoid serpins secreted by venom show phenoloxidase (PO) inhibitory activity, including venom serpin Lb-SPN in *Leptopilina boulardi* (Colinet et al. 2009), Pp-serpin1O in *Pteromalus puparum* (Yan et al. 2017), Cc-serpinB4 from *Cotesia chilonis* (Teng et al. 2022), and 2 newly reported venom serpins, *Microplitis mediator* MmvSPN-1 and MmvSPN-2 (Zhou et al. 2023). In fact, gene duplication is as common in insects as in vertebrates, and the diversity of serpins in *D. melanogaster* and *B. mori* is associated with gene duplication events (Garrett et al. 2009; Zhao et al. 2012). Proteomic analysis of venom apparatuses in parasitoid *M. mediator* also revealed widespread gene duplication of parasitoid serpins (Lin et al. 2019). However, there is insufficient evidence to show that gene duplication events cause neofunctionalization of serpins.

*Cotesia vestalis* (Hymenoptera: Braconidae) is a primary natural enemy of *Plutella xylostella* (Lepidoptera: Plutellidae), a worldwide migratory pest that attacks almost all cruciferous crops and causes high economic losses every year (Furlong et al. 2013; Li et al. 2016b). This host-parasitoid system is called *P. xylostella*-*C. vestalis* system. In addition to PDVs and venom, *C. vestalis* releases substantial amounts of strongly secretory teratocytes into the host hemocoel (Gao et al. 2016), which provides a splendid model for the study of the elaborate parasitic strategies on their hosts and parasitoid–host adaptive coevolution. In this study, we identified 25 *serpins* in *C. vestalis* based on the genome data (Shi et al. 2019). According to the phylogenetic analysis, most Cv-*serpin*s arose from gene duplication. Further positive selection analysis showed that the residue around the P1–P1′ cleavage sites of the RCL was under significantly positive selection. Notably, in addition to the 2 characterized serpins (Gu et al. 2021, 2022), 8 more serpins are teratocyte-specific and highly expressed, which leads us to ask whether the functions of these serpins are conserved and how the biological importance of serpins is represented in the parasitic lifestyle of *C. vestalis*. Our results showed that all these serpins show inhibitory activities and play multiple physiological roles in the host-parasitoid system. Intriguingly, in addition to suppression of the host melanization reaction (CvT-serpin1, 16, 18, and 21), we discovered that teratocyte serpins evolved new functions in defense against infection by pathogenic bacteria (CvT-serpin3 and 5) and modulated the host trehalose and triglyceride levels (CvT-serpin8 and 10).

## Results

### Characterization of *C. vestalis* Teratocyte Serpins

**Fig. 1.**
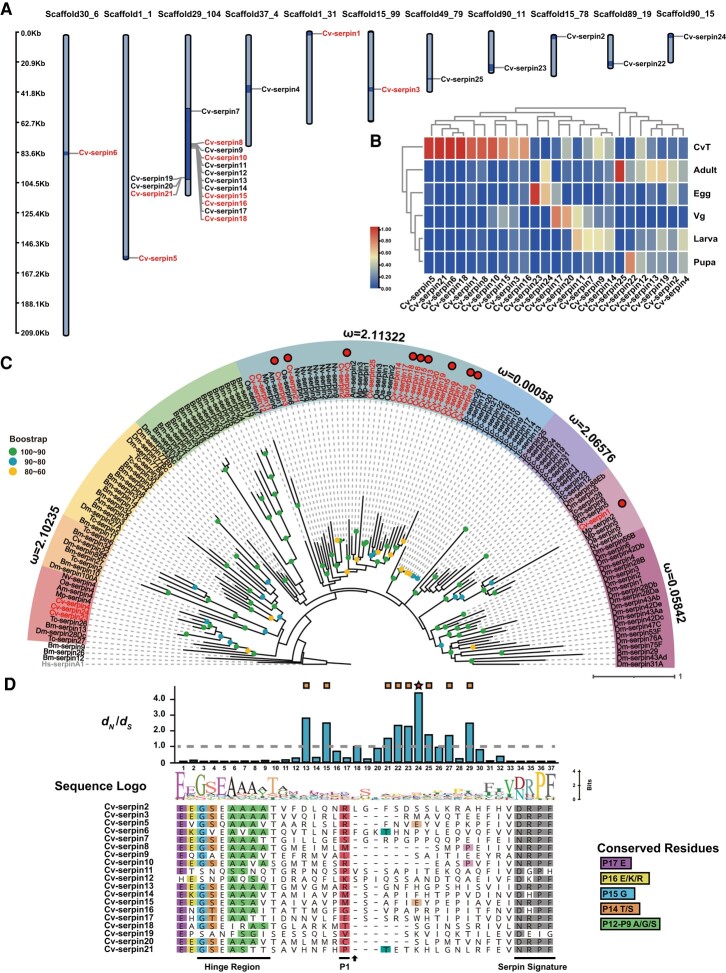
Phylogenetic relationship and positive selection of *C. vestalis* serpins. (A) The distribution of serpin genes in the *C. vestalis* genome. Above each bar, the scaffold numbers are displayed. The scale on the left side represents the size of the scaffolds. Additionally, serpins specially expressed in teratocytes are colored. (B) Expression profiles of *C. vestalis* serpin genes in different developmental stages and tissues. Log_2_ FPKM values for the serpins are presented by bar colors where the darker red represents higher expression values, the darker blue represents lower expression values. CvT, teratocytes of *C. vestalis*; Vg, venom gland of *C. vestalis*. (C) Phylogenetic relationship of Cv-serpins with other insects. Serpin genes from different insect taxonomy were chosen to construct a phylogenetic tree using the Maximum Likelihood (ML) method with *Homo sapiens* Hs-serpin1 as an outgroup. The resulting phylogenetic tree exhibits 9 different clades highlighted in distinct colors. Additionally, the dN/dS (ω) values are labeled aside each clade, and the values of ω > 999 (means ω = ∞) are hidden. The bootstrap values are indicated with dots of different colors on each branch node. Cv-serpin genes are specially marked in “red”, and teratocytes specifically expressed serpins are additionally highlighted with striking red circles. The bar right down the tree represents the tree scale. The first 2 letters in each of the serpins represent the abbreviation of the scientific name for a given species: Cv, *C. vestalis*; Nv, *N. vitripennis*; Oa, *O. abietinus*; Mp, *M. pharaonis*; Bm, *B. mori*; Dm, *D. melanogaster*; Am, *A. mellifera*; Tc, *T. castaneum*; Hs, *H. sapiens*. (D) Positive selection of Cv-serpins at the RCL region. The site model in CODEML was utilized to estimate sites under positive selection within the RCL of 19 serpin genes. Up: The bar plot above the sequence provides a visual representation of the dN/dS (ω) values. The number below the bar plot represents the site number of the sequence below. Star, site under significant positive selection (*P* > 0.99); square, site under positive selection (*P* > 0.75). Middle: The sequence logo indicates the relative frequencies of different amino acids in the sequence alignment. Down: the sequence alignment of the RCL of 19 serpin genes. The hinge and serpin signature regions of *C. vestalis* serpins are underlined. Conserved residues of the hinge region and serpin signature are highlighted in different colors in the sequence alignment. Predicted P1 residues are highlighted in red and the cleavage site between P1 and P1′ was marked with an arrow. The P4′ site of Cv-serpin5, 6, and 8 is respectively marked in different color, and the common amino acid at positive selection sites of other aligned serpins is marked with the same color.

We first identified 25 serpin genes spread across 11 different scaffolds in total from the *C. vestalis* genome (Shi et al. 2019). Two serpin clusters were found on scaffold29_104 (Fig. 1A): 1 cluster contained *Cv-serpin8-18* distributed in a range of 32 kb, and the other cluster included *Cv-serpin19-21* distributed in a range of 8 kb. These *Cv-serpins* were tandem repeats with high similarity, suggesting that they might be derived from gene duplication. The transcriptomes of 4 different developmental stages (egg, larva, pupa, and female adult) of *C. vestalis* samples and 2 tissue samples (venom gland and teratocytes) were previously sequenced in our laboratory (Gao et al. 2016; Zhao et al. 2017; Zhou et al. 2021). FPKM analyses showed that the expression pattern of *Cv-serpins* could be roughly divided into 2 groups (Fig. 1B). Interestingly, apart from the 2 characterized serpins (Gu et al. 2021, 2022), 8 additional serpins are teratocyte-specific with high expression and thus were named “*C. vestalis teratocyte serpins* (*CvT-serpins*)”.

We then obtained the full-length cDNA sequence of the 8 serpin genes, including *CvT-serpin1*, *CvT-serpin3*, *CvT-serpin5*, *CvT-serpin8*, *CvT-serpin10*, *CvT-serpin16*, *CvT-serpin18*, and *CvT-serpin21*. The length of the open reading frames of the *CvT-serpins* ranged from 1,011 to 1,359 bp, encoding polypeptides ranging from 404 to 463 amino acids (aa) with predicted molecular weights and theoretical isoelectric points ranging from 37.2 to 50.2 kDa and 5.86 to 9.62, respectively (Table 1). *CvT-serpin1*, *CvT-serpin8*, *CvT-serpin10*, *CvT-serpin18*, and *CvT-serpin21* were predicted to contain signal peptides. Although *CvT-serpin3*, *CvT-serpin5*, and *CvT-serpin16* had no predicted signal peptide, their secretion index ranged from 0.554 to 0.789, which is higher than the threshold value of 0.5 (Petitot et al. 2016), suggesting that they might be secreted extracellularly in a nonclassical way (Table 1). Secondary and tertiary structure analysis revealed that the 8 CvT-serpins comprised 10 to 11 α-helices, 3 β-sheets, and an RCL (supplementary fig. S1, Supplementary Material online).

**Table 1:** Protein characteristics of *C. vestalis* teratocyte serpins

Gene name	GeneBank number	Nucleotide length (bp)	Molecular weight (kDa)	Deduced amino acid residue	Isoelectric point (pH)	Signal peptide position	NN-score	P1–P1′cleavage site
CvT-Serpin1	OQ594896	1,359	50.2	453	6.294	Met1-Gly23	-	Arg407-Ser408
CvT-Serpin3	OQ594898	1,137	43.5	379	5.597	-	0.789	Lys329-Arg330
CvT-Serpin5	OQ594900	1,011	37.2	330	6.93	-	0.554	Arg294-Phe295
CvT-Serpin8	OQ594903	1,218	45.2	405	5.91	Met1-Ala17	-	Met366-Ser367
CvT-Serpin10	OQ594905	1,224	46.2	407	9.52	Met1-Ser21	-	Arg370-Glu371
CvT-Serpin16	OQ594911	1,311	49.1	437	6.21	-	0.618	Gly372-Ser373
CvT-Serpin18	OQ594913	1,209	45.9	402	9.62	Met1-Ala20	-	Thr364-Ser365
CvT-Serpin21	OQ594916	1,218	46.3	405	6.36	Met1-Ser17	-	Pro363-Thr364

### Gene Duplication and Adaptive Evolution of Cv-serpins

Phylogenetic analysis of Cv-serpins along with serpin family genes from other insects was constructed and dN/dS (ω) ratios were evaluated across branches using CODMEL under the branch model. The topology of the phylogenetic tree showed 19 of the 25 Cv-serpins were classified into 1 clade, and it has a higher ω value (ω = 2.11322) than other branches (Fig. 1C), suggesting possible positive selection in this clade.

Positive selection of specific functional sites in gene functional regions is an indispensable driving force to shape novel niches following gene duplication (Thomas et al. 2005; Qian et al. 2017; Zhu et al. 2019; Yang et al. 2020; Jovanovic et al. 2021). The RCL of serpins extends outwards from the protein surface and acts as a decoy for its homologous proteases, determining serpin activity and specificity (Meekins et al. 2017). To ascertain whether there is a positive selection, dN/dS values of the RCL sequences from the Cv-serpins clade (red circle marked) were calculated using the CODEML tools under site models. As a result, 9 sites close to the cleavage site had a probability > 75% for positive selection with dN/dS > 1, among which one residue around the P1 cleavage site of the RCL region was under significant (*P* > 0.99) positive selection (Fig. 1D, supplementary table S1, Supplementary Material online). A similar result was also detected according to the FEL method (supplementary table S2, Supplementary Material online). These results suggest that the sequence diversity of Cv-serpins is driven by both gene duplication and positive selection. As the RCL is a key region in the interaction between serpin and target proteases, the positive selection of residues around P1–P1′ cleavage sites may have a significant effect on the functional specificity of serpin.

Furthermore, we constructed a maximum likelihood tree based on the RCL amino acid sequences of CvT-serpins and serpins with known functions (supplementary fig. S2, Supplementary Material online). Notably, CvT-serpin1 shared 90% identity with MmvSPN-1, a parasitoid serpin suppressing host prophenoloxidase (PPO) activation by targeting HacSP29 (Zhou et al. 2023). CvT-serpin21 was clustered with serpins involved in modulating melanization such as CvT-serpin6, Pp-serpin1O, Of-serpin3, Ag-serpin2, and Dm-serpin27A (Ligoxygakis et al. 2002; Chu et al. 2015; Yan et al. 2017; Zhang et al. 2021b; Gu et al. 2022). In addition, the clade of (CvT-serpin3, CvT-serpin5, CvT-serpin15, CvT-serpin16, CvT-serpin18) was clustered with both anti-melanization and antimicrobial serpins (Song et al. 2008; Jiang et al. 2009; Pan et al. 2009; An et al. 2012; Lee et al. 2015; Liu et al. 2015a; Gu et al. 2021; Morrow et al. 2021; Teng et al. 2022; Zhou et al. 2023). CvT-serpin8 and 10, whose RCL sequences were clustered in the same branch of human alpha-1 antitrypsin, suggest that they might be related to metabolic regulation (Rabekova et al. 2021; O’Brien et al. 2022). Given the close association between serpin target proteases and RCL sequences, we speculated that CvT-serpins may function somewhat analogously to clustered serpins, and the functional diversity of CvT-serpins may extend beyond immune regulation.

### Inhibitory Activity of Recombinant CvT-serpins on Serine Proteases

**Fig. 2.**
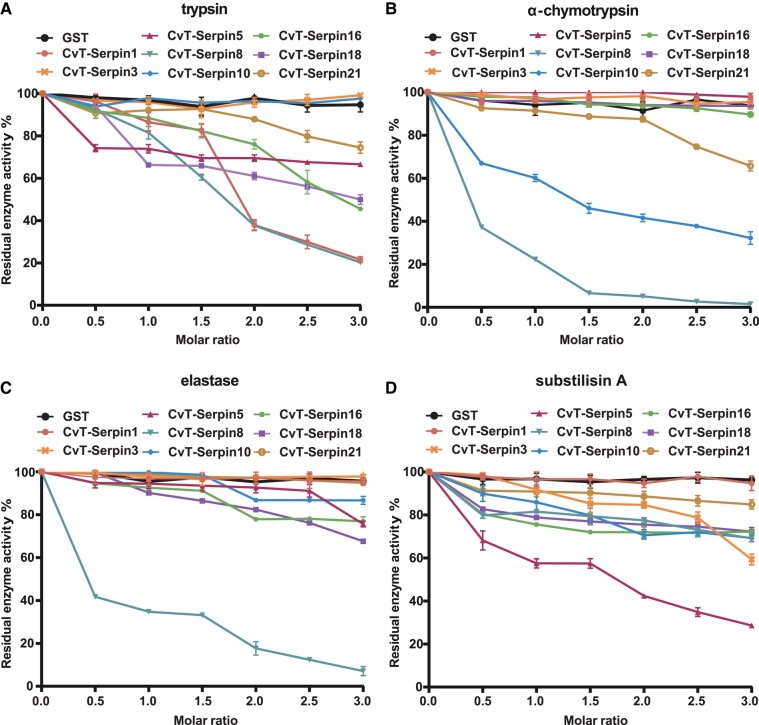
Inhibitory activities of rCvT-serpins on the activities of commercial serine proteases: trypsin (A), α-chymotrypsin (B), elastase (C), and subtilisin A (D). Different concentrations of each rCvT-serpin were separately mixed with these proteases with an increasing molar ratio of 0.5:1 to 3:1 in 100 mM Tris-HCl, pH 8.0, for 10 min at room temperature. Purified GST protein was used as a negative control, and an equal volume of PBS was used as a blank control. One unit of activity was defined as 0.001 ΔA_405_/min. The inhibitory effect of rCvT-serpins was plotted as the residual activity of proteases against the molar ratio of rCvT-serpins/proteases. Error bars represent the mean ± SD (*N* = 3).

To determine the activity of CvT-serpins, the N-terminal GST-tagged recombinant CvT-serpins protein was ectopically expressed in *Escherichia coli* and further purified. On SDS‒PAGE, each purified rCvT-serpin showed a single band with an estimated molecular weight ranging from 65 to 75 kDa, which corresponded to the predicted molecular weight (supplementary fig. S3, Supplementary Material online). Thereafter, the inhibitory effect of rCvT-serpins on 4 commercial proteases was tested (Fig. 2). As expected, when the molar ratio of GST/serpin to protease increased from 0.5 to 3.0, GST had no inhibitory activity on the 4 commercial proteases, whereas each rCvT-serpin showed strong (>60%), moderate (30% to 60%) or weak (<30%) inhibitory effects on the activities of the selected proteases (Fig. 2A to 2D). rCvT-serpin1, rCvT-serpin8, and rCvT-serpin10 displayed strong dose-dependent inhibition of 1 or 2 of the major digestive enzymes of insects, including trypsin, chymotrypsin, and elastase (Srinivasan et al. 2006; Zhan et al. 2011). rCvT-serpin16, 18, and 21 also had different degrees of a dose-dependent inhibitory effect on trypsin. Interestingly, most of the *Plutella xylostella* Clip serine proteases that participate in the PPO activation pathway possess the conserved trypsin-like serine protease (Tryp_Spc) domain (Lin et al. 2015), and the inhibitory effects of the above rCvT-serpins may indicate a potential regulatory role in PPO activation. In addition, the restraining effect on bacteriogenic subtilisin A of rCvT-serpin3 and rCvT-serpin5 infers their possible bacteriostatic function.

### Expression Profiles and Secretion of CvT-serpins

**Fig. 3.**
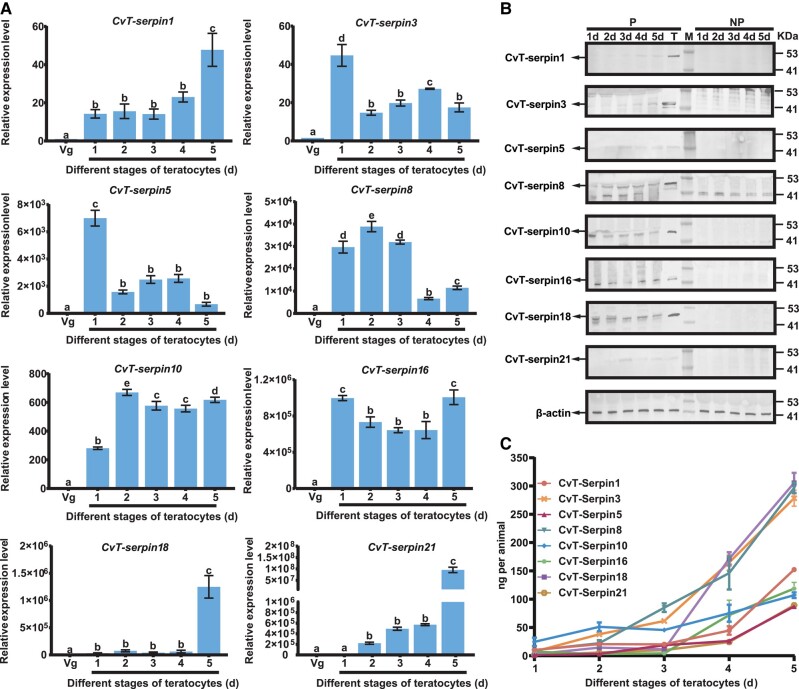
The expression and protein level of CvT-serpins. (A) The expressional pattern of CvT-serpins gene in venom glands (Vgs) and different developmental stages of teratocytes. (B) The protein level of CvT-serpins secreted by teratocytes. CvT-serpins were detected by immunoblotting at different developmental stages of teratocytes using the antibody against CvT-serpins. The target protein bands are marked with an arrow. Lane: M, protein marker; P, parasitized host larvae by *C. vestalis*; T, lysates of *C. vestali*s teratocytes; NP, nonparasitized host larvae by *C. vestalis*, β-actin was used as an internal reference. (C) Quantitative analysis of secreted CvT-serpins. CvT-serpins in plasma of parasitized *P. xylostella* at the different developmental stages of teratocytes were detected by ELISA (*N* = 672 for 1 d, *N* = 540 for 2 d, *N* = 458 for 3 d, *N* = 416 for 4 d, *N* = 338 for 5 d). Average CvT-serpin secretion levels were calculated for parasitized and nonparasitized *P. xylostella* larvae based on the number of samples per treatment. Values represent the means ± SD of 3 independent experiments (Tukey's test, bars labeled with different letters are significantly different at *P* < 0.05).

To study the expression dynamics of *CvT-serpins*, we tested the transcriptional levels of *CvT-serpins* in teratocytes aged from 1 to 5 d (the teratocytes at 60 h post-parasitization are defined as 1 d old) and the venom glands of 3-d-old mating females of *C. vestalis* by quantitative PCR (qPCR). The results showed that the transcriptional levels of *CvT-serpin 5* and *8* were higher in the early developmental stage (1 and 2 d old) of teratocytes. The transcript levels of other CvT-serpins were higher at the later development stage (4 and 5 d old) of teratocytes. None of the 8 serpins were detected in the venom glands, confirming that these serpins were uniquely expressed in teratocytes (Fig. 3A).

To explore whether serpin could be secreted into the extracellular space, we collected the cell-free hemolymph of *P. xylostella* 60 to 204 h after parasitism (i.e. 1 to 5-d-old teratocytes). By using a rabbit anti-CvT-serpin polyclonal antibody, we detected obvious protein bands in the hemolymph of *P. xylostella* after parasitism. The band sizes ranged from approximately 40 to 50 kDa, which corresponded to the predicted molecular weights (Table 1), and the concentration of serpin proteins in the hemolymph of *P. xylostella* increased with the development of teratocytes. However, no bands were detected in the nonparasitized *P. xylostella* (Fig. 3B and supplementary fig. S4, Supplementary Material online), suggesting that CvT-serpin proteins were secreted by *C. vestalis* teratocytes into *P. xylostella* hemolymph.

We also determined the content of CvT-serpins in the hemolymph of *P. xylostella* at different developmental stages of teratocytes by ELISA. The results showed that the content of all 8 serpins increased with the development time of teratocytes and peaked in 5-d-old teratocytes (Fig. 3C). Specifically, the content of the above CvT-serpins in *P. xylostella* can be divided into a high-content group including CvT-serpins 3, 8, and 18 with a content of more than 300 ng/insect and a low-content group including CvT-serpins 1, 5, 10, 16, and 21 with a content of less than 150 ng/insect.

### Inhibition of *P. xylostella* PPO Activation by rCvT-serpin1, 16, 18, and 21

**Fig. 4.**
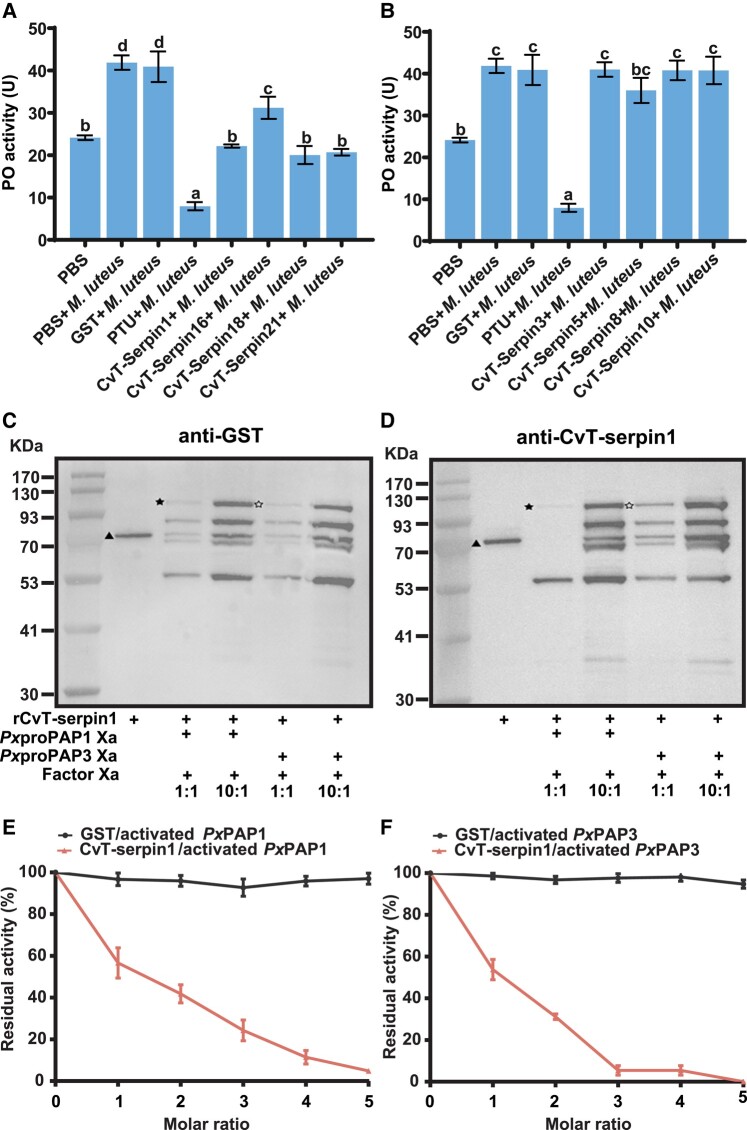
Inhibition of *P. xylostella* PPO activation by rCvT-serpins. (A and B) Inhibition of PPO activation of *P. xylostella* hemolymph by rCvT-serpins. The amount of enzyme that increased absorbance by 0.001 per minute (ΔA_490_) was defined as 1 unit of PO activity. Hemolymph mixed with PBS was used as blank controls, mixtures using the same volume of PBS to replace rCvT-serpins were used as negative controls, and mixtures with saturated PTU were used as positive controls. Error bars represent the mean ± SD (*N* = 6). The data were conducted 1-way analysis of variance (1-way ANOVA, Tukey’s test, *P* < 0.05). Significant differences are indicated with different letters. (C and D) Detection of covalent complex formation between rCvT-serpin1 and activated *Px*PAP1 or *Px*PAP3 by immunoblot analysis using antiserum against GST (C) or rCvT-serpin1 (D). The complexes were detected by immunoblotting using antibodies against mouse anti-GST tag (1:2,000) or rabbit anti-CvT-serpin (1:500) as the primary antibodies and HRP-conjugated anti-rabbit/mouse IgG (1:5,000) as the secondary antibody. In the control samples, *Px*proPAP1Xa or *Px*proPAP3Xa was superseded with an equal volume of reaction buffer. The sizes and positions of molecular mass standards are indicated on the left of each blot. Solid star, rCvT-serpin1/activated *Px*PAP1; hollow star, rCvT-serpin1/activated *Px*PAP3 complex; triangle, rCvT-serpin1. (E and F) Stoichiometry for inhibition of activated *Px*PAP1 and *Px*PAP3 by rCvT-serpin1. One unit of amidase activity was defined as ΔA_405_/min = 0.001. The residual IEARase activity of *Px*PAP1 or *Px*PAP3 was plotted as mean ± SD (*N* = 3) against the corresponding molar ratios of rCvT-serpin1 and *Px*PAP1 or *Px*PAP3. An equal quantity of GST protein was used as a negative control.

To date, insect serpins have been widely found to target serine proteases involved in the phenoloxidase (PO) cascade, which regulates the melanization of hemolymph. Therefore, we first investigated the effect of CvT-serpins on the PPO activation of *P. xylostella*. Four serpins, rCvT-serpin1, 16, 18, or 21, showed a strong inhibitory effect on the PO activity of *P. xylostella* with reductions of 47.1%, 25.5%, 52.2%, and 50.6%, respectively (Fig. 4A), while other rCvT-serpins did not inhibit the *P. xylostella* PO activity (Fig. 4B).

In *P. xylostella*, *Px*PAP1 and *Px*PAP3, as terminal proteases in the PO cascade, participate in PPO cleavage and activation (Lin et al. 2015). To test whether *Px*PAPs could be regulated by CvT-serpins, we first assessed whether a covalent protein complex formed between active *Px*PAP1 or *Px*PAP3 and purified rCvT-serpins (supplementary fig. S5, Supplementary Material online). The results showed that when Factor Xa-activated *Px*proPAP1Xa or *Px*proPAP3 was mixed with rCvT-serpin1, the intensity of the 75 kDa band corresponding to rCvT-serpin-1 decreased, and a new band appeared at approximately 110 kDa, the expected size of the covalent complex of rCvT-serpin1 and *Px*PAP1 or *Px*PAP3. As the molar ratio of rCvT-serpin-1:PAP1Xa/PAP3Xa was increased from 1:1 to 10:1, the band became more intense. Meanwhile, some nonspecific bands were also observed, possibly due to the degradation of the rCvT-serpin1 or rCvT-serpin1-activated *Px*PAP1/*Px*PAP3 complex by Factor Xa (Fig. 4C and 4D). However, no corresponding composite bands were observed after Factor Xa-activated *Px*proPAP1_Xa_ and *Px*proPAP3_Xa_ coincubated with rCvT-serpin16, 18, or 21 (supplementary fig. S6, Supplementary Material online). These results indicated that CvT-serpin1 could form complexes with activated *Px*PAP1 and *Px*PAP3, while the other 3 CvT-serpins may target other serine proteases in the PO cascade.

To explore the potential inhibition of *Px*PAP1 and *Px*PAP3 by CvT-serpin1, the IEARase activity of *Px*PAP1 and *Px*PAP3 was tested in the presence of rCvT-serpin1. The IEARase activity of *Px*PAP1/*Px*PAP3 decreased linearly as the CvT-serpin1 concentration increased (Fig. 4E and 4F). The inhibitory stoichiometries to *Px*PAP1 and *Px*PAP3 were approximately 4.0 and 3.0, respectively. This result suggested that CvT-serpin1 preferentially acts as an inhibitor rather than a substrate of *Px*PAP1 and *Px*PAP3 under our experimental conditions.

### Antimicrobial Activity of rCvT-serpin3 and rCvT-serpin5

**Fig. 5.**
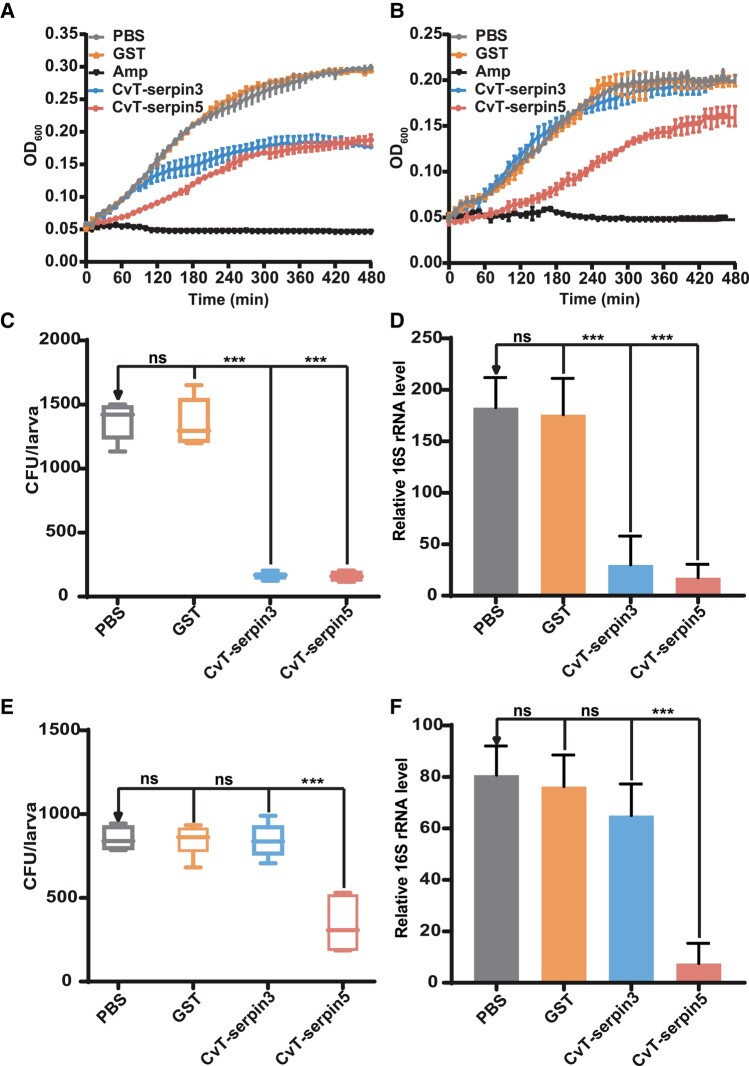
Antimicrobial activity of rCvT-serpin3 and rCvT-serpin5. (A and B) The growth curves of *S. aureus* (A) and *E. coli* (B) after rCvT-serpin3 or rCvT-serpin5 treatment, respectively. Twenty microliters of protein solution containing 30 μg rCvT-serpin3 or 10 μg rCvT-serpin5 was added into 80 μl bacterial suspension. The mixtures were incubated at 37 °C and the absorbance at 600 nm was recorded every 10 min. PBS, GST, and 1 mg/ml of ampicillin were used as the blank, negative, and positive controls, respectively. Error bars represent the mean ± SD (*N* = 3). (C and D) Bacterial loads in *P. xylostella* larvae after *S. aureus* infection. Early fourth instar *P. xylostella* larvae were coinjected *S. aureus* with rCvT-serpin3 (300 ng/insect) or rCvT-serpin5 (100 ng/insect). Bacterial load level was determined by colony forming units (CFU) assay (C) and relative 16S rRNA level (D). (E and F) Bacterial loads in *P. xylostella* larvae after *E. coli* infection by CFU assay (E) and relative 16S rRNA level (F). For the CFU assay (C and E), the boxes show the first to third quartiles and the median, and the whiskers show the range (Mann–Whitney, *P < 0.05; **P < 0.01; ***P < 0.001; ns, not significant; *N* = 10 to 12 from 6 to 8 independent experiments). For the qPCR assay (D and F), *Px-β-Actin* and *Px-β-tubulin* were used as internal references, and experiments were performed with at least 6 independent replicates, using at least 10 *P. xylostella* larvae for each replicate. Values represent the mean ± SD of 6 independent experiments (Tukey's test, *P < 0.05; **P < 0.01; ***P < 0.001; ns, not significant).

The above inhibitory activity tests confirmed the inhibitory activity of rCvT-serpins 3 and 5 against bacteriogenic subtilisin A, thus, we investigated whether these *CvT-*serpins have antimicrobial activity. First, we examined the inhibitory effect of the rCvT-serpins on the growth of the gram-positive bacterium *Staphylococcus aureus* and the gram-negative bacterium *E. coli* in vitro. Compared with the negative control, the growth of *S. aureus* was significantly suppressed by both rCvT-serpin3 and rCvT-serpin5 from 180 and 120 min post-treatment (Fig. 5A), and the growth of *E. coli* was significantly suppressed by rCvT-serpin5 from 100 min post-treatment (Fig. 5B). Nevertheless, there was no significant difference in the growth of *S. aureus* or *E. coli* between the other 6 rCvT-serpin-treated groups and the negative control group (supplementary figs. S7 and S8, Supplementary Material online). We further determined the antibacterial activity of rCvT-serpin3 and rCvT-serpin5 in *P. xylostella* larvae. Both the colony forming units (CFU) counts and the 16S rRNA level were significantly reduced after coinjection of rCvT-serpin3 or rCvT-serpin5 with *S. aureus* in *P. xylostella* larvae (Fig. 5C and 5D). The coinjection of rCvT-serpin5 significantly suppressed the bacterial load of *E. coli* in *P. xylostella* larvae, while the coinjection of rCvT-serpin3 had no significant effect (Fig. 5E and 5F). These results suggested that CvT-serpin3 and 5 may act as immune effectors that participate in the innate immune response of *P. xylostella*-*C. vestalis* system.

### CvT-serpins Manipulate Immune Homeostasis in the *P. xylostella*-*C. vestalis* System

**Fig. 6.**
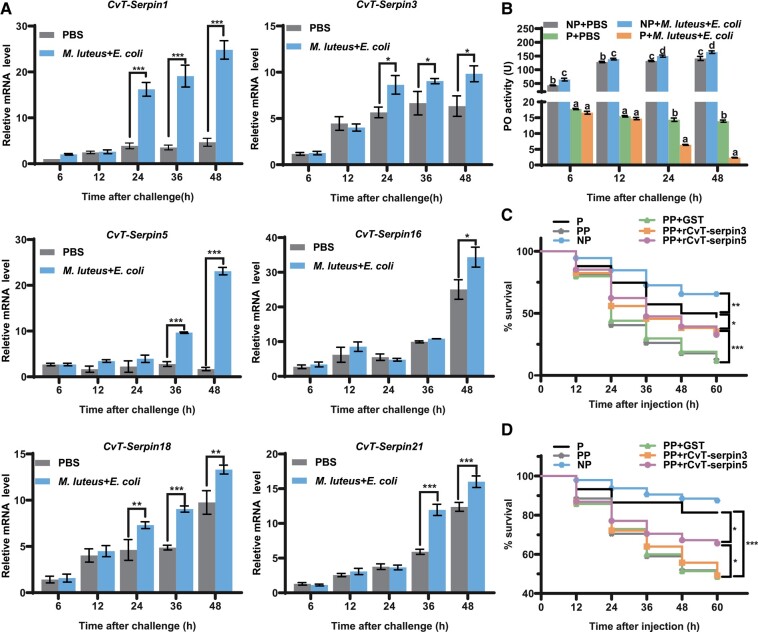
The effects of CvT-serpins on immune homeostasis in the *P. xylostella*-*C. vestalis* system. (A) Expression pattern of *CvT-serpins* in teratocytes in vivo following microbial challenges. *P. xylostella* larvae at 5 d post-parasitization were challenged with an equimolar mixture of inactivated *E. coli* and *S. aureus*, and then teratocytes were collected and subjected to qPCR analysis. PBS was used as the negative control. Values represent the mean ± SD of 3 independent experiments (Tukey's test, *P < 0.05; **P < 0.01; ***P < 0.001). (B) Hemolymph PO activity of parasitized and nonparasitized *P. xylostella* larvae after microbial challenge. PBS was used as the negative control. Error bars represent the mean ± SD (*N* = 6). Significant differences were indicated with different letters (1-way ANOVA, Tukey’s test, *P* < 0.05). (C and D) The survival of *P. xylostella* larvae following *S. aureus* (C) or *E. coli* (D) challenges. Experiments were performed with 3 independent replicates, using at least 30 *P. xylostella* larvae for each replicate. Differences between groups were analyzed by the log-rank test (Mantel–Cox, *P < 0.05; **P < 0.01; ***P < 0.001).

As 6 of the 8 CvT-serpins were involved in immune responses, we asked how these serpins respond to immune challenges. Thus, we detected the transcriptional levels of the 8 CvT-serpins after being challenged by an equimolar mixture of 2 different bacteria, gram-positive *S. aureus* and gram-negative *E. coli*. The results showed that the transcriptional levels of all tested CvT-serpins were upregulated at 48 h post-infection (hpi), which suggested that CvT-serpins may play an important role in the immune response of the *P. xylostella*-*C. vestalis* system (Fig. 6A).

Consistent with our previous results, the melanization reaction of *P. xylostella* was significantly suppressed due to parasitism (Fig. 6B) (Wang et al. 2021b). Notably, in contrast to the induction effect of foreign pathogen infection in the unparasitized host, PO activity was significantly reduced in parasitized hosts 24 to 48 hpi compared with the PBS-treated group. The reduction rate reached nearly 99% at 48 hpi in comparison with that in the unparasitized hosts.

We further investigated the effect of immune challenges on the survival of the host. To exclude the influence of endogenous CvT-serpins, we included a group of pseudoparasitized *P. xylostella* when treating *P. xylostella* with *S. aureus* or *E. coli*. As shown in Fig. 6C and 6D, the survival rate of both parasitized and pseudoparasitized *P. xylostella* was significantly lower than that of nonparasitized *P. xylostella* after *S. aureus* injection, and pseudoparasitized *P. xylostella* had the lowest survival rate. When *S. aureus* was coinjected with rCvT-serpin3 or rCvT-serpin5 into the pseudoparasitized hosts, the survival rates were significantly increased in comparison to those treated with no rCvT-serpins (Fig. 6C). A similar trend was observed after *E. coli* infection, except for parasitized *P. xylostella* (Fig. 6D). The coinjection of rCvT-serpin5 with *E. coli* significantly improved the survival of pseudoparasitized *P. xylostella* larvae, while the coinjection of rCvT-serpin3 had no such effect. These results indicated that teratocyte-mediated immune responses are crucial for the survival of both *P. xylostella* and *C. vestalis*.

### rCvT-serpin8 and rCvT-serpin10 Modulate Trehalose and Triglyceride Levels in *P. xylostella*

**Fig. 7.**
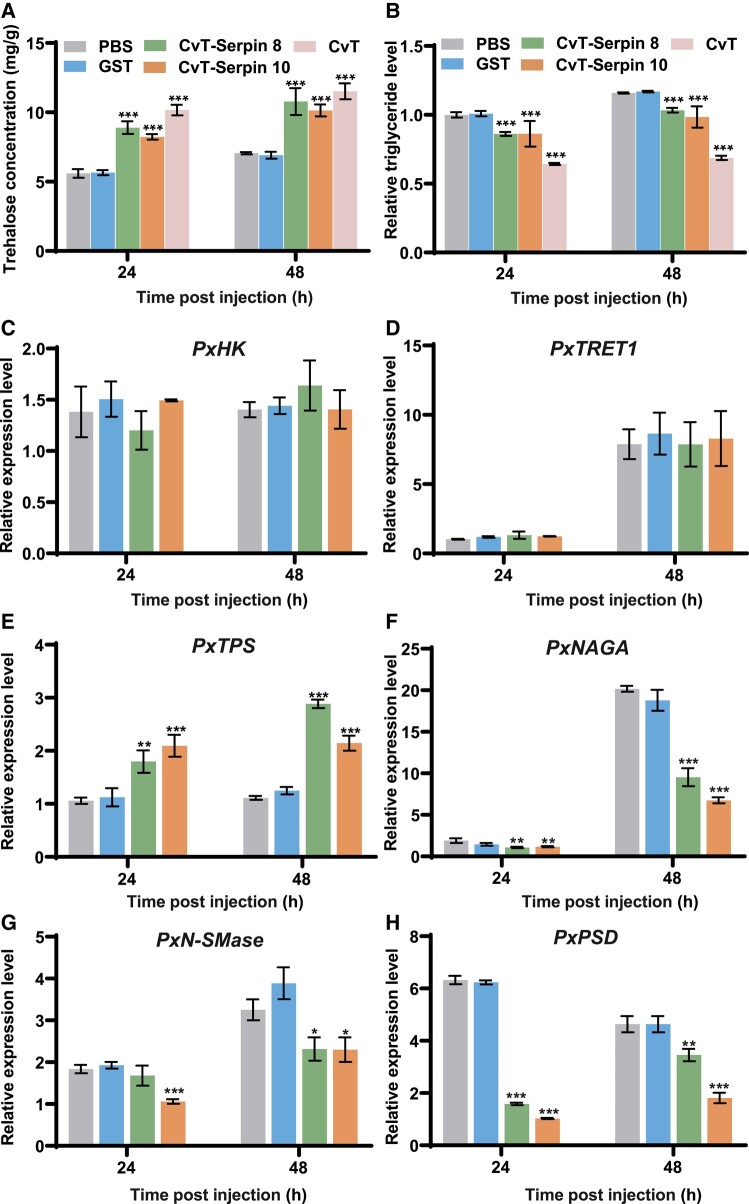
rCvT-serpin8 and rCvT-serpin10 modulate the trehalose and triglyceride levels of *P. xylostella*. (A) The effects of rCvT-serpin8 and 10 on the trehalose content of *P. xylostella*. (B) The effects of rCvT-serpin8 and 10 on the triglyceride content of *P. xylostella.* Teratocytes’ content (CvTC), GST, and PBS were used as positive, negative, and blank controls, respectively. (C to H) The effects of rCvT-serpin8 and 10 on the expression of genes associated with trehalose metabolism (*PxHK*, *PxTPS*, and *PxTRET1*) and triglyceride metabolism (*PxNAGA*, *PxN-SMase*, and *PxPSD*). HK, hexokinase; TPS, trehalose-6-phosphate synthase; TRET1, trehalose transporter 1; NAGA, alpha-N-acetylgalactosaminidase; N-SMase, neutral sphingomyelinase; PSD, phosphatidylserine decarboxylase proenzyme. Values represent the mean ± SD of 6 biological replicates (Tukey's test, *P < 0.05; **P < 0.01; ***P < 0.001).

Our previous work suggested that teratocytes play an important role in the reduction of host systemic lipid levels in *P. xylostella* after *C. vestalis* parasitization (Wang et al. 2021a). Accordingly, we wondered whether CvT-serpins participate in regulating trehalose and triglyceride levels in *P. xylostella.* We injected early fourth instar *P. xylostella* larvae with the highest amount of each CvT-serpin calibrated by ELISA. Then, we determined the contents of trehalose and triglyceride in the hemolymph of the treated *P. xylostella* larvae at 24 and 48 hpi. We found that the trehalose levels were dramatically increased in the *P. xylostella* larvae microinjected with rCvT-serpin8 or rCvT-serpin10 at 24 and 48 hpi (Fig. 7A), while the triglyceride levels were markedly decreased with the injection of rCvT-serpin8 or rCvT-serpin10, which showed a similar trend to the positive control group injected with CvTC (Fig. 7B). In contrast, the remaining 6 rCvT-serpins showed no regulation of trehalose and triglycerides (supplementary fig. S9, Supplementary Material online).

To further explore the effects of rCvT-serpin8 and rCvT-serpin10 on trehalose and triglyceride metabolic pathways, we detected the transcripts of 3 genes (*PxHK*, *PxTPS*, and *PxTRET1*) involved in trehalose synthesis and 3 genes (*PxNAGA*, *PxN-SMase*, and *PxPSD*) related to lipid metabolism after the injection of rCvT-serpin8 or rCvT-serpin10 (Fig. 7C to 7H). Compared with the levels in the negative and blank controls, there was a marked increase in *PxTPS* transcriptional levels and a decrease in *PxNAGA*, *PxN-SMase*, and *PxPSD* transcriptional levels when treated with rCvT-serpin8 or rCvT-serpin10, while the transcription levels of *PxHK* and *PxTRET1* seemed not to be affected. These results inferred that CvT-serpin8 and CvT-serpin10 may be involved in the regulation of proteins related to the above metabolic pathways, thus regulating the content of trehalose and triglycerides in the host.

## Discussion

As the largest family of serine proteinase inhibitors, serpins are widespread in all kingdoms, playing key roles in multitudinous physiological and phytochemical pathways, including inflammation, immunomodulation, tumorigenesis, hemolymph coagulation, PPO activation, antimicrobial activity, and cancer metastasis (Heit et al. 2013; Meekins et al. 2017; Schemczssen-Graeff et al. 2021; Zhang et al. 2021a). However, scientific understanding of the functional diversity of insect serpins is very limited. In this study, we characterized 25 *C. vestalis* serpins, most of which resulted from gene duplication, and found that the residues around the P1–P1′ cleavage sites of the RCL region were under positive selection. We further filtered out 8 *C. vestalis* teratocytes secreted serpins for functional analysis. The results showed that CvT-serpin1, CvT-serpin16, CvT-serpin18, and CvT-serpin21 maintain a conservative function in PPO activation inhibition as with other insect species, while the other 4 serpins exhibited functional differentiation; CvT-serpin3 and CvT-serpin5 evolved antibacterial ability, and CvT-serpin8 and CvT-serpin10 were neo-functionalized to regulate the host's trehalose and triglyceride metabolism.

Serpin inhibitory functions are usually dictated by the RCL amino acid sequence (Huntington et al. 2000). The residue adjacent to the P1 cleavage site in the RCL, particularly amino acid spanning from P4 to P4′, is highly variable between diverse serpins and is regarded as a major determinant of the inhibition specificity of serpins (Gomes et al. 2014; Sanrattana et al. 2021). Our positive selection site determination showed that the fierce selection around the P1–P1′ cleavage site shapes the change in its amino acids (Fig. 1D) and the experimentally confirmed function differentiation and neofunctionalization of CvT-serpins could be attributed to this change. We discovered a potential connection between the selection site and function divergence by comparing multiple alignments of the Cv-serpin RCL sequences that clustered in 1 lineage: except for CvT-serpin3 and CvT-serpin18, CvT-serpin5 and CvT-serpin15 with antibacterial property shared Glu at the P4′ site, while metabolic regulatory factor CvT-serpin8 and CvT-serpin10 shared Pro at the same selected site. We also found that the serpin regulating melanization shared the same residues at the selected functional sites, for instance, CvT-serpin6 shared Thr with CvT-serpin21 at the P4′ site. However, further study is warranted to unlock the true relationship between specific RCL positions and function diversity. Consistent with the results of positive selection analysis and multiple sequence alignment, a phylogenetic tree based on RCL sequences of 10 CvT-serpins and other serpins with known functions demonstrated a possible correlation between RCL and serpin functions (supplementary fig. S2, Supplementary Material online). Combined with the fact that most *CvT-serpins* may be derived from gene duplication, these discoveries suggest that the multiple differentiation of CvT-serpin functions may be caused by the adaptive evolution of amino acids in functional regions on its RCL.

Phenol oxidase–mediated melanization, one of the most important innate immune defenses of insects against microbial or parasitic infestation, is catalyzed by a series of proteolytically activated serine proteases and is controlled by protease inhibitors from the serpin superfamily (Wang et al. 2020b). It is widely recognized that parasitic wasps use serpins as conserved weapons to inhibit host PPO activation, for instance, Lb-SPN, *Cotesia chilonis* Cc-serpinB4, Cc-ESSPNDP, Pp-serpin1O, MmvSPN-1, and MmvSPN-2 (Colinet et al. 2009; Yan et al. 2017; Teng et al. 2022; Zhou et al. 2023). As the key enzyme in the PO cascade, PPO is present in the hemolymph in an inactive state and requires proteolytic cleavage to transform into its active form phenoloxidase (PO), which is often carried out by a trypsin-like serine protease (PAP or PPAE), and the activated PO catalyzes the generation of quinones which further transformed into melanin to encapsulate and eliminate invaders (Zhang et al. 2022). The inhibitory activity assay revealed that rCvT-serpins 1, 16, 18, and 21 displayed inhibitory effects on trypsin activity in a dose-dependent manner (Fig. 2A), suggesting that these CvT-serpins were much more likely to participate in PPO activation inhibition. Consistently, we found that those 4 serpins significantly repressed PPO activation in *P. xylostella* (Fig. 4A). Sequentially, we obtained activated *Px*PAP1 and *Px*PAP3 (supplementary fig. S5, Supplementary Material online) in vitro, and a follow-up experiment proved that rCvT-serpin1 inhibited activated *Px*PAP1 and *Px*PAP3 by forming SDS-stable complexes in a dose-dependent manner (Fig. 4C and 4D). Indeed, our previous research has shown that rCvT-serpin6 could also form SDS-stable complexes of serpin-protease with *Px*PAP1 and *Px*PAP3 (Gu et al. 2022) with disparate inhibitory efficiency. The inhibitory stoichiometry of rCvT-serpin1 was approximately 4 for *Px*PAP1 and 3 for *Px*PAP3 (Fig. 4E and 4F) compared with that of 5 for *Px*PAP1 and 4 for *Px*PAP3 of rCvT-serpin6 (Gu et al. 2022). Both results suggested that CvT-serpin1 may display a higher inhibitor ability for *Px*PAP than CvT-serpin6. However, we detected no complexes of CvT-serpin16, 18, or 21 coincubated with activated *Px*PAP1 and *Px*PAP3 (supplementary fig. S6, Supplementary Material online), suggesting that they failed to regulate *Px*PAP1 and *Px*PAP3. Considering that the PPO activation pathway inhibited by serpin is a complicated network mediated by multifarious hemolymph serine proteases as well as noncatalytic serine protease homologues (SPHs), we speculate that CvT-serpin16, 18, and 21 are likely to be potential inhibitors of other serine proteases involved in the PPO activation network.

Several crustacean serpins from *Penaeus monodon*, *Litopenaeus vannamei*, and *Marsupenaeus japonicus* exhibit an inhibitory role in the growth of bacteria or fungi (Homvises et al. 2010; Somnuk et al. 2012; Zhao et al. 2014; Liu et al. 2015b, 2016, 2017). MjSerp1, a serpin from *M. japonicus*, was demonstrated to inhibit the activity of the microbial serine proteases subtilisin A and proteinase K and thus suppress the growth of bacteria (Zhao et al. 2014). However, there is little evidence that insect serpins have antibacterial properties. In our previous work, we identified an antibacterial serpin secreted by *C. vestalis* teratocytes, CvT-serpin15 (Gu et al. 2021). In this work, 2 more CvT-serpins, CvT-serpin3 and CvT-serpin5, were found to inhibit bacterial growth both in vitro and in vivo, of which rCvT-serpin3 showed a suppressive effect on *S. aureus* and rCvT-serpin5 had effects on both *S. aureus* and *E. coli* (Fig. 5). The inhibitory activity tests showed that these 2 serpins were able to inhibit the activity of subtilisin A, which is derived from a gram-positive bacterium, *Bacillus licheniformis* (Fig. 2D). Combining the antibacterial effects and inhibitory activity, we speculated that these 2 serpins infer bacterial growth possibly by inhibiting bacterial secreted serine proteases.

When developing in the host's hemocoel, parasitic wasp larvae encounter a variety of survival challenges, one of the most fatal aspects of which is the infection of pathogenic microorganisms, while a series of immune defenses of the host, including the melanization response, is suppressed after parasitism (Anderl et al. 2016; Wang et al. 2021b). An immune challenge assay demonstrated that bacterial infection could induce the expression of *CvT-serpins* in parasitized *P. xylostella* larvae (Fig. 6A). The PO activity of bacteria-infected parasitized hosts was significantly decreased compared with that of the negative control, which was the opposite of the induction effects on unparasitized *P. xylostella* larvae (Fig. 6B). As 4 of them were demonstrated to inhibit the activation of the PO cascade, we assumed that the unexpected inhibition of PO activity in parasitized *P. xylostella* larvae was mediated by CvT-serpins, by which the parasitic wasps avoid their offspring being subjected to the damage of excessive melanization caused by bacterial infection after parasitization. Thus, it is reasonable to observe a lower survival rate in parasitized hosts than in unparasitized hosts. The different mortality rates between parasitized and pseudoparasitized larvae and the treatment of antibacterial serpins, CvT-serpin3 or CvT-serpin5, suggested that CvT-serpins act as an immune defense factor in *P. xylostella*-*C. vestalis* system. Based on these results, our findings demonstrated that CvT-serpins participate in the reprogramming of the host immune system through sustained inhibition of host melanization while simultaneously producing other immune effectors to compensate for this suppression.

Sugar and lipid metabolism are important components of insect nutrition metabolism (Kafsack and Llinás 2010; Shukla et al. 2015; Zhou et al. 2022). Although several vertebrate serpins have been proven to participate in sugar and lipid metabolism, no similar functions were reported in invertebrate serpins. Interestingly, our results ascertained that CvT-serpin8 and CvT-serpin10 could upregulate trehalose and reduce triglyceride levels in *P. xylostella* by affecting the transcripts of genes involved in the trehalose and triglyceride metabolic pathways (Fig. 7A and 7B). As shown in previous work, the enhancement of host trehalose content as well as the downregulation of host lipid content are beneficial to the growth and development of wasp offspring (Kumar et al. 2016; Wang et al. 2021a). The regulation of trehalose and triglyceride metabolism by serpins is a novel and ingenious strategy by which parasitic wasps regulate host homeostasis. However, the deeper regulatory mechanism remains to be further explored.

**Fig. 8.**
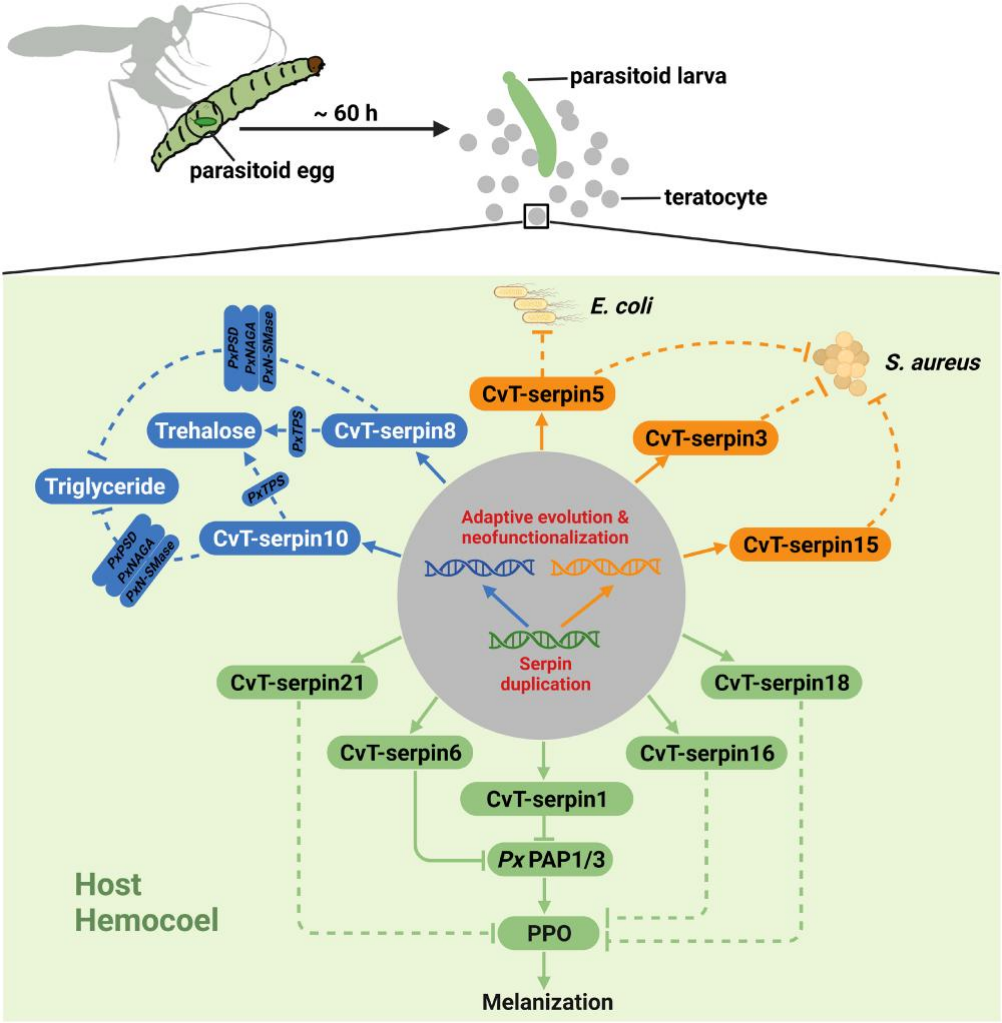
Schematic representation of *C. vestalis* teratocytes serpins in manipulating parasitized *P. xylostella* larvae homeostasis via adaptive evolution and neofunctionalization. After parasitism, *C. vestalis* teratocytes dissociated from the embryonic cellular membrane and released into the hemocoel of the host as the egg hatched. Some of the genes containing the serpin domain in teratocytes underwent a series of adaptive gene replication events ultimately leading to functional innovation. Once expressed and secreted into the hemolymph of the *P. xylostella* larvae host, these serpins differentiated multifunction: CvT-serpin1, 6, 16, 18, and 21 showed insect-conserved serpin function of inhibiting the PPO activation in host hemolymph, CvT-serpin3, 5, and 15 evolved bacteriostasis to compensate for the weakened immunity of parasitized *P. xylostella* larvae, and CvT-serpin8 and 10 differentiated neofunction into regulate host nutrient metabolism by increasing the trehalose level and decreasing the triglyceride level. All these functions guarantee homeostasis for the development of parasitoid larvae.

Recent works revealed that parasitoid venom serpins achieved phenotypic diversity and ecological adaptation to parasitic microenvironment via both gene duplication and alternative splicing from an evolutionary standpoint, suggesting that parasitoid serpins are emerging as a classic example for uncovering the arms race between host and parasite (Spence et al. 2021; Yan et al. 2023; Ye et al. 2023). Our findings further provided evidence that gene duplication and positive selection enabled CvT-serpins to evolve novel functions, e.g. bacteriostasis and nutrient metabolism regulation, in addition to their conserved function in melanization inhibition. Consequently, the reprogramming of the host immune system and manipulation of host carbohydrate metabolism mediated by CvT-serpins will facilitate the survival and development of parasitoids’ offspring (Fig. 8). This study not only provides a noticeable case of neofunctionalization in tandems of *C. vestalis* teratocyte-specific serpins but also deepens our insights into parasite–host coevolution: The long-term arms race with the host puts strong evolutionary pressure on parasitic wasps to evolve new weapons through various strategies to gain advantages in their interactions. Furthermore, the prospect of selective manipulation of serpin function may help to open a new avenue to progress treatment means for metabolic disorders and infectious diseases.

## Materials and Methods

### Insects

Endoparasitoid *C. vestalis* and its host *P. xylostella* were reared as previously described (Wang et al. 2018). In brief, *P. xylostella* was fed cabbage at 25 °C and 65% relative humidity under a 14:10 light:dark cycle. Adult *P. xylostella* and *C. vestalis* were fed with a 20% honey/water (V/V) solution. To obtain parasitized host larvae, middle third instar *P. xylostella* larvae were exposed to a single *C. vestalis* female wasp that had eclosed for 2 d and mated adequately in a finger tube with a diameter of 10 mm and a height of 70 mm. Pseudoparasitization was conducted by cobalt-60-irradiated female wasps as described in our previous work (Wang et al. 2021a). In this case, the irradiated female wasps were able to inject venom and PDV into the host hemocoel, but the eggs were unable to hatch, so no teratocytes were produced.

### Sample Collection

*Cotesia vestalis* teratocytes (CvT), venom glands (Vgs), and hemolymph were collected following previously described methods (Wang et al. 2018). For teratocyte collection, the parasitized host was dissected to release wasp larvae, teratocytes, and hemocytes from the hemocoel into a serum-free medium (Thermo Fisher Scientific, USA). After the hemocytes adhered to the bottom of the dish (30 min), the larger free teratocytes were collected and transferred to another dish with a fresh medium. The above steps were repeated 5 times to ensure that there was no contamination of any host hemocytes or tissue debris in the obtained teratocytes. After centrifugation at 500 × *g* at room temperature for 5 min, the supernatant was discarded, and teratocytes were used for further study. For the injection of teratocyte contents, 5-d-old teratocytes were collected from the hemocoel of 100 *P. xylostella* larvae and resuspended in 10 μl of PBS followed by transfer to a grinding tube for crushing. After centrifugation of the flinders at 4 °C for 10 min, the supernatant was collected and frozen at −80 °C for subsequent injection. Each *P. xylostella* larva was injected with 0.1 μl of teratocyte contents (CvTC), which is the number of teratocytes contained in each parasitized host.

### Phylogenetic Analysis, Positive Selection, and Protein Structure Prediction

The sequences of CvT-serpin genes and their deduced amino acid sequences were analyzed by DNASTAR software 9.02 (Wisconsin, USA), and all obtained CvT-serpins sequences were analyzed by SMART (Letunic et al. 2015) to confirm the existence of the serpin domain. Signal peptides were predicted with SignalP 5.1 (http://www.cbs.dtu.dk/services/SignalP/), and the NN-score was predicted with SecretomeP 2.0 (http://www.cbs.dtu.dk/services/SecretomeP/). The P1–P1′ cleavage site in the CvT-serpin amino acid sequence was predicted by the MEROPS Peptidase Database (https://www.ebi.ac.uk/merops/). The tertiary protein structures of CvT-serpins were predicted with the AlphaFold2 Protein Structure Database (Jumper et al. 2021) and visualized using PyMOL v1.7.0.0 (https://pymol.org/2/).

In the phylogenetic analysis of serpin genes, serpin genes from 4 other Hymenoptera species (*Apis mellifera*, *Nasonia vitripennis*, *Orussus abietinus*, and *Monomorium pharaonis*), a Diptera species (*Drosophila melanogaster*), a Lepidoptera species (*Bombyx mori*), and a Coleoptera species (*Tribolium castaneum*) were retrieved from public genome data (supplementary table S3, Supplementary Material online). Totally, a set of 148 serpin sequences were aligned using the Multiple Alignment using Fast Fourier Transform (MAFFT) v7.480 software (Katoh and Standley 2013). To remove randomized sequence sections in the multiple sequence alignments, Alicut v2.31 (https://github.com/PatrickKueck/AliCUT) was employed. The ModelFinder tool in the IQ-Tree v2 package (Kalyaanamoorthy et al. 2017) was utilized to predict the best model for phylogenetic analysis. Subsequently, the phylogenetic tree was constructed based on the VT+G4 model by IQ-Tree v2 (Minh et al. 2020) with parameters “-bb 1000” and “-alrt 1000”. The phylogenetic tree was used as an input treefile to assess the dN/dS value (model = 0 and model = 2) across branched using the branch model in the CODEML tools of PAML v4.9j (Yang 2007; Álvarez-Carretero et al. 2023). The results were then visualized using iTOLs (https://itol.embl.de/), allowing for clear visualization and interpretation of the phylogenetic relationships among the serpin genes.

To detect positive selection sites in the clustered *C. vestalis* serpins, sequences of the RCL region were aligned using MUSCLE 5.1. linux64 software (Edgar 2004). Subsequently, dN/dS values were calculated using the site model (model = 0, NSsites = 7, and model = 0, NSsites = 8) in the CODEML tools of PAML v4.9j, employing the Bayes Empirical Bayes (BEB) method (Yang 2007; Álvarez-Carretero et al. 2023). This approach allows the differentiation of sites undergoing positive selection from those evolving neutrally or experiencing purifying selection. In addition, positive selection at individual sites was also inferred using the FEL method with default parameters at https://www.datamonkey.org (Kosakovsky Pond and Frost 2005).

### Gene Cloning and Expression Profile Analyses

Total RNA was isolated from collected teratocytes, venom glands of *C. vestalis* or the whole bodies of treated *P. xylostella* larvae using TRIzol agent (Vazyme, China) and reverse-transcribed into cDNA using the PrimeScript 1st Strand cDNA Synthesis Kit (Takara, Japan). The entire coding regions of *CvT-serpin1*, *3*, *5*, *8*, *10*, *16*, *18*, and *21* were cloned and sequenced. Primer sequences are listed in supplementary table S4, Supplementary Material online.

For qPCR, total RNA was extracted and then reverse-transcribed into cDNA using the ReverTra Ace qPCR RT Kit (Toyobo, Japan). The qPCRs were implemented using Thunderbird qPCR Mix (Toyobo, Japan) on the CFX Connect Real-Time System (Bio-Rad, USA). qPCRs were performed for 60 s at 95 °C, followed by 40 cycles of 15 s at 95 °C and 30 s at 60 °C. Both *Cv*-*18S rRNA* and *Cv*-*β-tubulin* of *C. vestalis* were used as internal reference genes for *C. vestalis*-derived genes, and both *Px-β-Actin* and *Px-β-tubulin* of *P. xylostella* were used as internal reference genes for *P. xylostella* genes. The relative expression levels were calculated using the 2^−ΔΔCt^ method. All the primers and the GenBank accession number of the corresponding gene used for qPCR in this study are displayed in supplementary table S4, Supplementary Material online.

### Production of Recombinant CvT-serpin Proteins and Antiserum Against CvT-serpins

The DNA fragments encoding mature *CvT-serpin1*, *3*, *5*, *8*, *10*, *16*, *18*, and *21* were amplified by PCR using the specific primers listed in supplementary table S4, Supplementary Material online and then subcloned into the pGEX6P-1 vector (Novagen, Germany) and transformed into the *E. coli* strain BL21 (DE3) after sequence confirmation. To express recombinant proteins, single clones were incubated in an LB medium containing 100 mg/ml ampicillin at 37°C. When the OD_600_ of the culture reached 0.8, the recombinant protein was expressed for 12 h at 22 °C and 200 × rpm with the addition of 0.5 mM isopropyl b-D1-thiogalactopyranoside (IPTG). The bacteria were harvested by centrifugation, resuspended in lysis buffer, and lysed by sonication. The cleared supernatant was obtained by centrifugation and used for purifying the soluble protein of recombinant CvT-serpins by Glutathione Resin (Genscript, China) according to the manufacturer's protocol. The eluted protein was analyzed by 12% SDS‒PAGE, visualized by staining with Coomassie blue, quantitated by the Bradford method, and stored at −80 °C for further use. To obtain antiserum against CvT-serpins, we selected the specific amino acid segment of each CvT-serpin to synthesize polypeptide as an antigen to immunize rabbits and produce rabbit polyclonal antibodies (ABclonal Tech, China) (supplementary table S5, Supplementary Material online).

### Inhibitory Activities of rCvT-serpins

The inhibitory activities of 8 rCvT-serpins were determined using 4 serine proteases, including trypsin (Sigma, USA), α-chymotrypsin (Sigma, USA), elastase (Sigma, USA), and subtilisin A from *B. licheniformis* (Sigma, USA), as previously depicted with wispy modifications (Gu et al. 2021). The residual protease activities were detected at 405 nm on a microplate reader (Thermo Fisher Scientific, USA) by the addition of 1 mM specific chromogenic substrates in 50 mM Tris-HCl buffer containing 50 mM NaCl and 5 mM CaCl_2_, pH 7.5, Nα-benzoyl-L-arginine 4-nitroanilide hydrochloride (Sigma B3133, USA) for trypsin, N-succinyl-Ala-Ala-Pro-Phe p-nitroanilide (Sigma S7388, USA) for α-chymotrypsin, N-succinyl-Ala-Ala-Pro-Leu p-nitroanilide (Sigma S8511, USA) for elastase, and Z-Gly-Gly-Leu p-nitroanilide (Sigma C3022, USA) for subtilisin A.

### Western Blot Analyses

Protein samples were mixed with 5 × SDS protein loading buffer (Sangon, China) and boiled for 10 min. Boiled proteins were separated by 12% SDS‒PAGE followed by transfer to a polyvinylidene difluoride (PVDF) membrane. After being blocked and washed, the membranes were incubated with primary antibodies against each CvT-serpin (1:500) or primary antibodies against β-actin (1:4,000, ABclonal, China, as an internal reference) overnight at 4 °C. Then, membranes were incubated with secondary antibody HRP-conjugated anti-rabbit/mouse IgG (1:5,000, ABclonal, China) for 1.5 h at room temperature. After being washed with PBST 5 times, the membranes were incubated with FDbio-Femto ECL Western blotting Substrate for imaging (Fdbio, China). To detect the recombinant protein with GST-tag or His-tag, the membranes were incubated with primary antibodies against the mouse anti-GST-Tag monoclonal antibody (1:2,000, ABclonal) or mouse anti-His-Tag monoclonal antibody (1:2,500, ABclonal, China), respectively.

### ELISAs

Secretory CvT-serpin levels in the hemolymph of parasitized and nonparasitized *P. xylostella* larvae were quantified by an ELISA kit (Solarbio, China) according to the manufacturer's protocol. Cell-free hemolymph was obtained as described above, diluted 4-fold in coating buffer and placed in 96-well coated plates at 100 μl/well overnight at 4 °C. Plates were washed 3 times with PBS containing Tween-20 (PBST) and blocked with PBS containing 2% BSA overnight at 4 °C. Plates were then washed with PBST before adding polyclonal antibodies against each CvT-serpin (1:200) at 37 °C for 2 h and then incubated for 1 h at 37 °C with 100 μl HRP-labeled goat anti-rabbit IgG (1:2,000 dilution with coating buffer). After thoroughly washing with PBST, 100 μl of TMB-ELISA substrate was added at 37 °C for 30 min, then the reaction was stopped by adding 50 μl/well of 2 M sulfuric acid, and absorbance was estimated at 450 nm immediately.

### PO Activity and Inhibitory Stoichiometry Assay

PO activity was tested as previously described (Gu et al. 2019). Twenty microliters of freshly acquired cell-free hemolymph (10 × diluted), 10 μl of rCvT-serpin (1 μg/μl), and 2 μl inactivated *M. luteus*, as an elicitor (0.5 μg/μl), were mixed at room temperature for 30 min, and then 100 μl L-dopamine was used as a substrate to measure the PO activity at ΔA_490_. And the serpin-protease complex formation assay was performed according to our previous study (Gu et al. 2022). In brief, purified recombinant *Px*proPAP1Xa (0.25 μg) and *Px*proPAP3Xa (0.25 μg) were activated by Factor Xa (0.25 μg) and mixed with the purified rCvT-serpins at a molar ratio of 1:1 or 10:1 (serpin:*Px*PAP) at room temperature for 30 min and then subjected to 12% SDS–PAGE. For the detection of inhibitory stoichiometry for rCvT-serpins against *Px*PAP1Xa or *Px*PAP3Xa, purified rCvT-serpin1 were incubated with the Factor Xa-activated *Px*proPAP1Xa or *Px*proPAP3Xa at different molar ratios in a volume of 20 μl of reaction buffer (20 mM Tris, 150 mM NaCl, 2 mM CaCl_2_, pH 8.0). The rCvT-serpin inhibitory stoichiometry for *Px*PAP1Xa and *Px*PAP3Xa was verified by determining the residual amidase activity of the reaction cocktail. An equal quantity of GST protein was used as a negative control. After incubation at room temperature for 30 min and the addition of 200 μl of the colorimetric substrate acetyl-Ile-Glu-Ala-Arg-p-nitroanilide (IEARpNA), the residual amidase activity of the reaction mixtures was measured by monitoring the absorbance at 405 nm.

### Bacterial Growth Curve Measurement

The antimicrobial activity of CvT-serpins in vitro was investigated according to the method reported previously (Wang et al. 2013). *S. aureus* or *E. coli* were grown to an OD_600_ of 0.8 at 37 °C in Mueller Hinton (MH) broth and then diluted to OD_600_ = 0.05 with PBS. Subsequently, the serially diluted bacterial strains were severally mixed with different rCvT-serpins to make the final concentrations of each rCvT-serpin in the mixtures reach the value measured by ELISA per insect (150 ng/μl for rCvT-serpin1, 300 ng/μl for rCvT-serpin3, 100 ng/μl for rCvT-serpin5, 300 ng/μl for rCvT-serpin8, 100 ng/μl for rCvT-serpin10, 100 ng/μl for rCvT-serpin16, 300 ng/μl for rCvT-serpin18, and 100 ng/μl for rCvT-serpin21). In the control groups, equal amounts of PBS, GST protein, and ampicillin (1 mg/ml) were added as blank, negative and positive treatments, respectively. The growth of bacteria was analyzed by measuring the absorbance of the above mixtures at 600 nm every other 10 min from 0 to 480 min.

### Bacterial Load and Survival Rate Assessment

To estimate the antimicrobial activity of CvT-serpins in vivo, 0.1 μl samples of equivalent mixtures of rCvT-serpin (300 ng/μl for rCvT-serpin3 and 100 ng/μl for rCvT-serpin5) with *S. aureus* (OD_600_ = 0.1) or *E. coli* (OD_600_ = 0.1) were injected into unparasitized early fourth instar *P. xylostella* larvae by a FemtoJet 4i Microinjector (Eppendorf, Germany) with a microcontroller (Narishige, Japan). An equal dose of PBS or GST protein was used as a blank or negative control, respectively. Injected *P. xylostella* larvae were then fed normally until 24 h post-injection, the surface of the treated larvae was disinfected with 75% alcohol, and then homogenized after the midgut was removed. This homogenate was then 100 times diluted with sterile PBS before being incubated on LB agar at 37 °C for 24 h, and then the number of CFU was counted. Synchronously, DNA was extracted from the above-treated larvae using FastPure Cell/Tissue DNA Isolation Mini Kit (Vazyme, China) to determine the relative levels of 16S rRNA by qPCR. For the survival rate analysis, pseudoparasitized, unparasitized, or parasitized early fourth instar *P. xylostella* larvae were injected in the same way as for bacterial load assessment. The injected *P. xylostella* larvae were reared at 25 °C and provided with fresh food daily, and the death rate was recorded every 12 h.

### Trehalose and Triglyceride Level Measurements

Early fourth instar *P. xylostella* larvae were injected with the highest amount of each CvT-serpin calibrated by ELISA. A total of 0.1 μl of teratocyte content (CvTC), 0.1 μl of 2 mg/ml GST, and 0.1 μl of PBS were injected separately as positive, negative, and blank treatments, respectively. Injected *P. xylostella* larvae were then fed normally until 24 and 48 h post-injection when the concentration of trehalose in the hemolymph and the total triglyceride content were measured, respectively. The trehalose levels in the hemolymph of *P. xylostella* larvae were detected according to a protocol described previously with slight modifications (Mayack et al. 2020). Briefly, the collected cell-free hemolymph was diluted 12-fold with acetonitrile (CH_3_CN) followed by centrifugation for 6 min at 10,000 × *g* at 4 °C, and the samples were retained on ice until filtered through a membrane filter with a pore size of 0.22 μm (Millipore, USA). The filtrate was used for HPLC analyses. Ten microliters of prepared samples were injected into an Agilent 1100 high-performance liquid chromatograph (Agilent Technologies, USA) and separated at 30°C constant temperature using a 5 μm Nucleosil-100 NH_2_ column (250 × 4 mm^2^, Knauer, Germany) with a flow rate of 1 ml/min. The concentration of trehalose in the hemolymph of *P. xylostella* larvae was then calculated using a standard curve obtained by running a standard solution of trehalose on the same column.

Triglyceride levels were detected as previously described (Wang et al. 2021a). The whole bodies of treated *P. xylostella* larvae were obtained and homogenized in PBS containing 0.1% Triton and then centrifuged for 10 min at 14,000 × rpm. After heat-inactivation at 70 °C for 5 min, the triglyceride levels were surveyed with serum TG determination kits (Sigma, USA).

### Statistical Analyses

All statistical analyses expected for survival and CFU analysis of *P. xylostella* larvae were implemented using SPSS 22.0 software by 1-way ANOVA and Tukey's test with a significance threshold of *P* < 0.05. Data are expressed as the mean ± standard deviation (SD). Significant values are indicated as *P < 0.05, **P < 0.01, and ***P < 0.001. Survival and CFU analysis were severally performed using the log-rank (Mantel–Cox) test and Mann–Whitney, with GraphPad Prism 8.

## Supplementary Material

msad269_Supplementary_Data

## Data Availability

The data underlying this article are available in the article and in its Supplementary material.
