# Supplementary material for: Parasitoid Serpins Evolve Novel Functions to Manipulate Host Homeostasis
Source: Mol Biol Evol. 2023 Dec 7;40(12):msad269. doi: 10.1093/molbev/msad269 (PMC10735303; doi:10.1093/molbev/msad269)
Supplement: msad269_Supplementary_Data [file msad269_supplementary_data.zip › Supplemental_figure_MBE-23-0474_final.pdf]

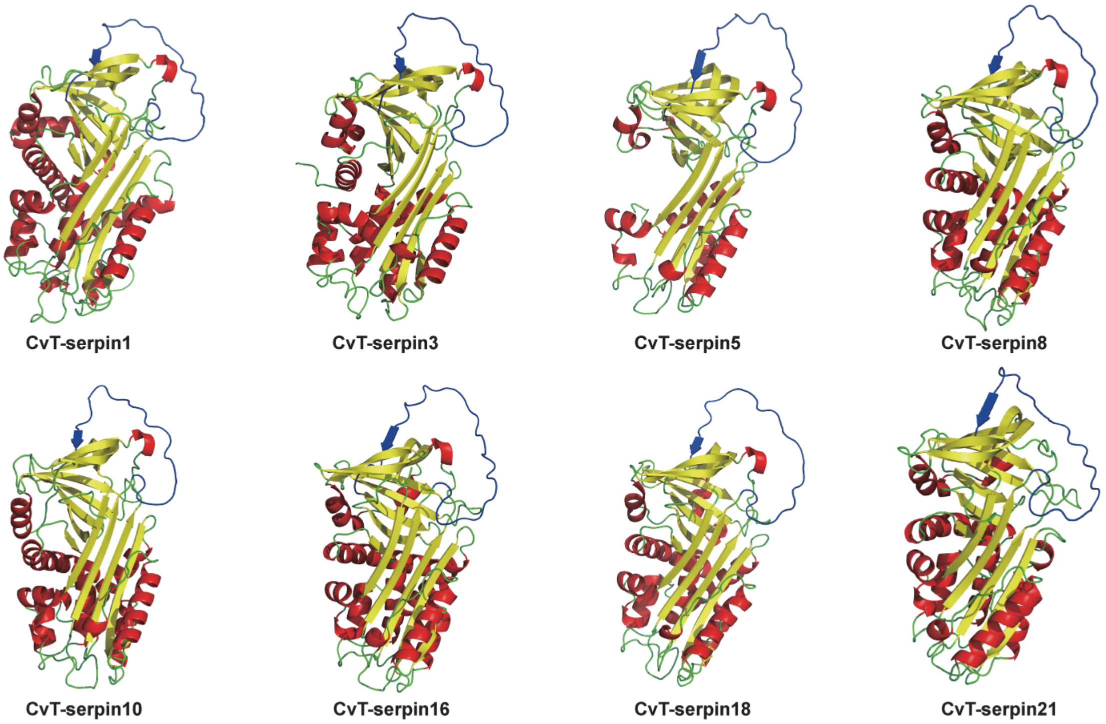

Figure S1. Predicted tertiary protein structures of CvT-serpins obtained through AlphaFold2. Red, yellow, and blue indicate α-helices, β-sheets and RCL domains, respectively.

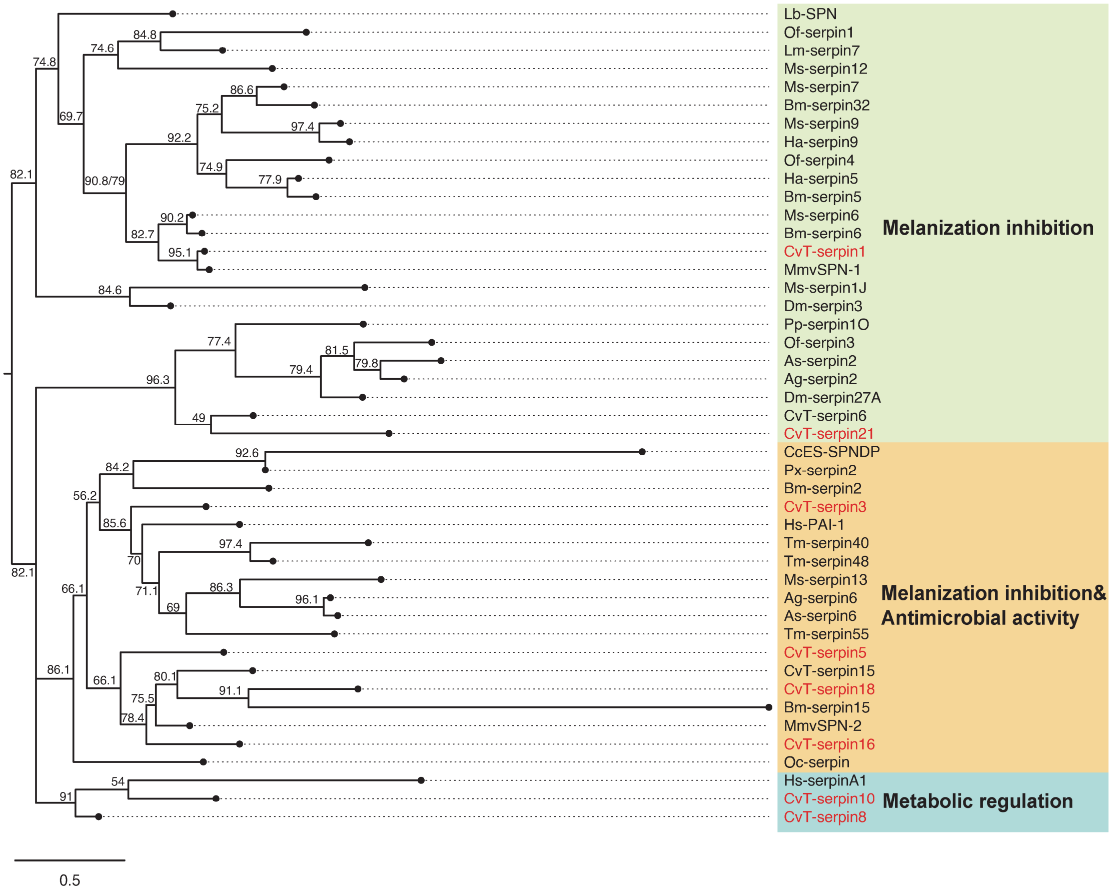

Figure S2. The maximum likelihood mid-point rooted tree of ten CvT-serpins and serpins from other species with known functions based on RCL amino acid sequence. The bootstrap was set as 1000 replicates. The number on the branch represents the bootstrap value. The serpins in red font on the tree represent the newly identified serpins in this study, while the rest are serpins of known functions. In each branch, different color blocks and bold fonts are used to roughly classify the functions of reported serpins from different species.

The GenBank accession number for sequences used in this phylogenetic analysis: Ms-serpin1J, Ms-serpin6, Ms-serpin7, Ms-serpin12, Ms-serpin13 (AAC47340.1, AAV91026.1, ADM86478.1, AYK02795.1, AYK02793.1, *Manduca sexta*); Bm-serpin2, Bm-serpin5, Bm-serpin6, Bm-serpin15, Bm-serpin32 (NP_001037021.1, NP_001037205.1, NP_001103823.1, NP_001139707.1, NP_001139723.1 *Bombyx mori*); Ag-serpin2, Ag-serpin6 (ABJ52801.1, ABJ52806.1, *Anopheles gambiae*); As-serpin2, As-serpin6 (KFB50806.1, KFB52518.1, *Anopheles sinensis*); Pp-serpin1O (AOW41297.1, *Pteromalus puparum*); Lb-

SPN (ACQ83466.1, *Leptopilina boulardi*); CcES-SPNDP (MW300024.1, *Cotesia chilonis*); MmvSPN-1, MmvSPN-2 (GGCK01004359.1, GGCK01005859.1, *Microplitis mediator*); Lm-serpin7 (QIR83414.1, *Locusta migratoria*); Hs-PAI-1 , Hs-serpinA1 (AAA60003.1, KAI2572614.1, *Homo sapiens*); Dm-serpin3, Dm-serpin27A (CAB63098.1, NP_652024.1, *Drosophila melanogaster*); Tm-serpin40, Tm-serpin48, Tm-serpin55 (BAI59106.1, BAI59108.1, BAI59107.1, *Tenebrio molitor*); Of-serpin1, Of-serpin3, Of-serpin4 (AFV46312.1, AHA43071.1, UTM04190.1, *Ostrinia furnacalis*); Ha-serpin5, Ha-serpin9 (ATD13319.1, ATD13320.1, *Helicoverpa armigera*); Oc-serpin (ALF44673.1, *Osmia cornifrons*) and Px-serpin2 (BAF36820.1, *Plutella xylostella*).

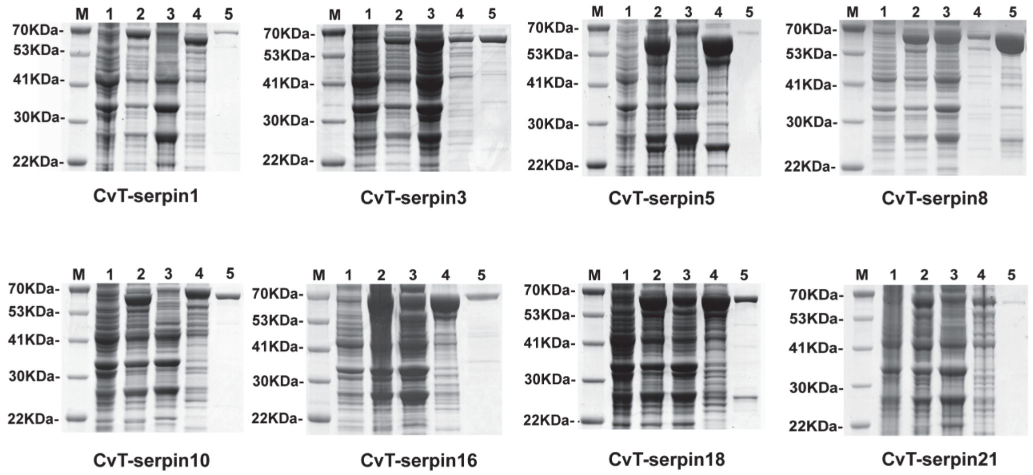

Figure S3. Analysis of prokaryotic expression and purification of recombinant CvT-serpins (rCvT-serpins) by 12% SDS-PAGE gels. M, protein marker; lane 1, non-induced *E. coli*; lane 2, induced *E. coli* with 0.5mM IPTG; lane 3, supernatant protein; lane 4, inclusion body; lane 5, purified rCvT-serpins.

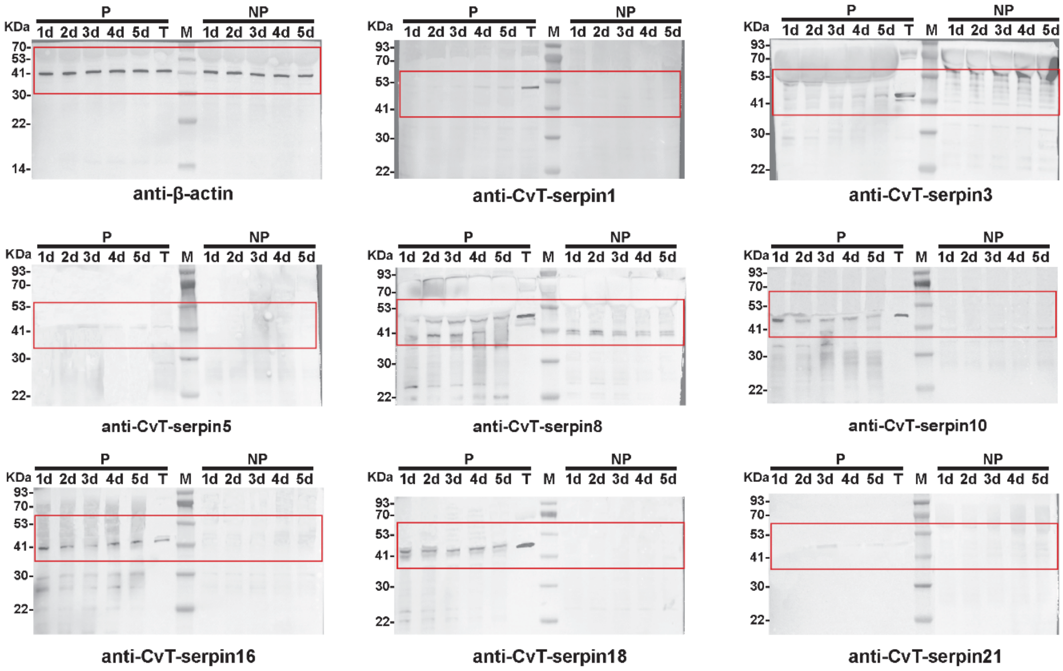

Figure S4. The full image of the Western blot used in Figure 3B. The protein level of CvT-serpins secreted by teratocytes. CvT-serpins were detected by immunoblotting at different developmental stages of teratocytes using the antibody against CvT-serpins. The target protein bands are marked with an arrow. Lane: M, protein marker; P, parasitized host larvae by *C. vestalis*; T, lysates of *C. vestalis* teratocytes; NP, non-parasitized host larvae by *C. vestalis*, β-actin was used as an internal reference.

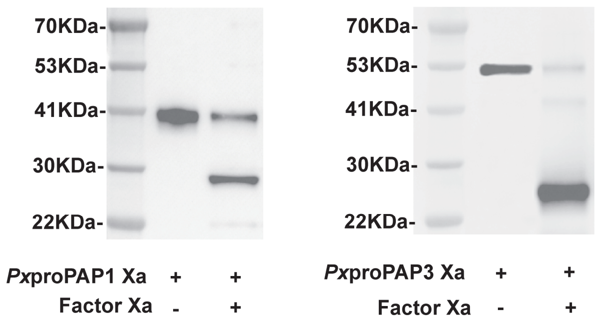

Figure S5. Activation of *Px*proPAP1Xa and *Px*proPAP3Xa. Purified recombinant *Px*proPAP1Xa and *Px*proPAP3Xa were activated by Factor Xa at 24 °C for 6 h. The Western blotting analysis was conducted using antiserum against His.

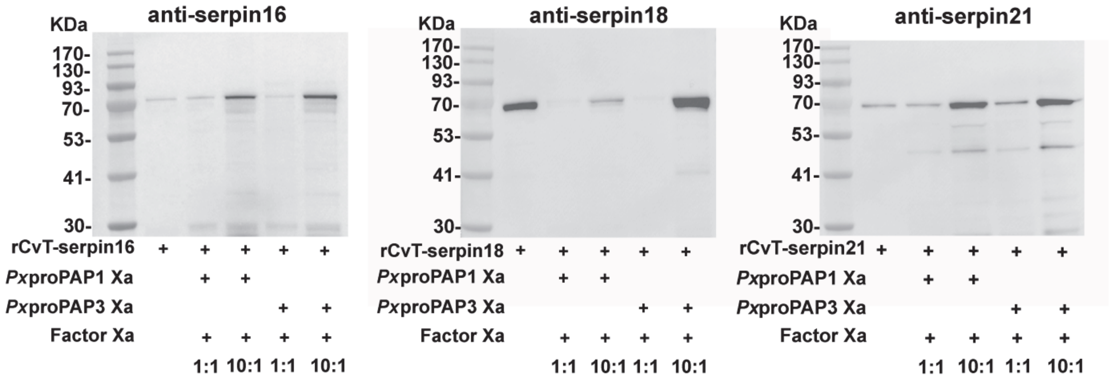

Figure S6. Covalent complex formation after Factor Xa-activated *Px*proPAP1Xa and *Px*proPAP3Xa being co-incubated with other CvT-serpin16, 18 and 21. Purified recombinant *Px*proPAP1Xa and *Px*proPAP3Xa were activated by Factor Xa as mentioned above and severally mixed with the purified rCvT-serpin16, 18, 21 at a molar ratio of 1:1 or 10:1 (serpin: *Px*PAP) at room temperature for 30 min and then subjected to 12% SDS-PAGE. The sizes and positions of molecular mass standards are indicated on the left of each blot. Complexes bands were detected using FDbio™ ECL Western Blotting Substrate and imaged by ChemiDoc MP Imaging System.

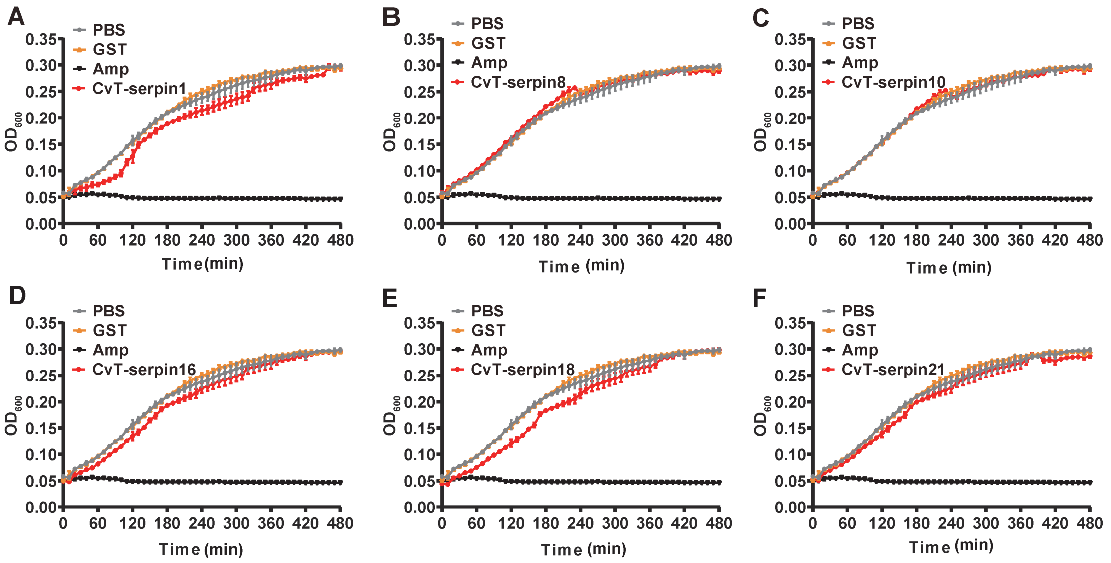

Figure S7. The growth curves of *Staphylococcus aureus* exposed to rCvT-serpin1 (A), rCvT-serpin8 (B),

rCvT-serpin10 (C), rCvT-serpin16 (D), rCvT-serpin18 (E), rCvT-serpin21 (F). *S. aureus* was cultured to exponential phase, and then diluted with fresh medium to OD_{600}=0.05. 20 µl protein solution contains 15 µg rCvT-serpin1, 30 µg rCvT-serpin8, 10 µg rCvT-serpin10, 10 µg rCvT-serpin16, 30 µg rCvT-serpin18 or 10 µg rCvT-serpin21 was added into 80 µl bacterial suspension. The mixtures were incubated at 37 °C and the absorbance at 600 nm was recorded every 10 min. PBS, GST, and 1 mg ml^{-1} of ampicillin was used as the blank, negative and positive controls, respectively. Error bars represent the mean ± S.D. (N = 3).

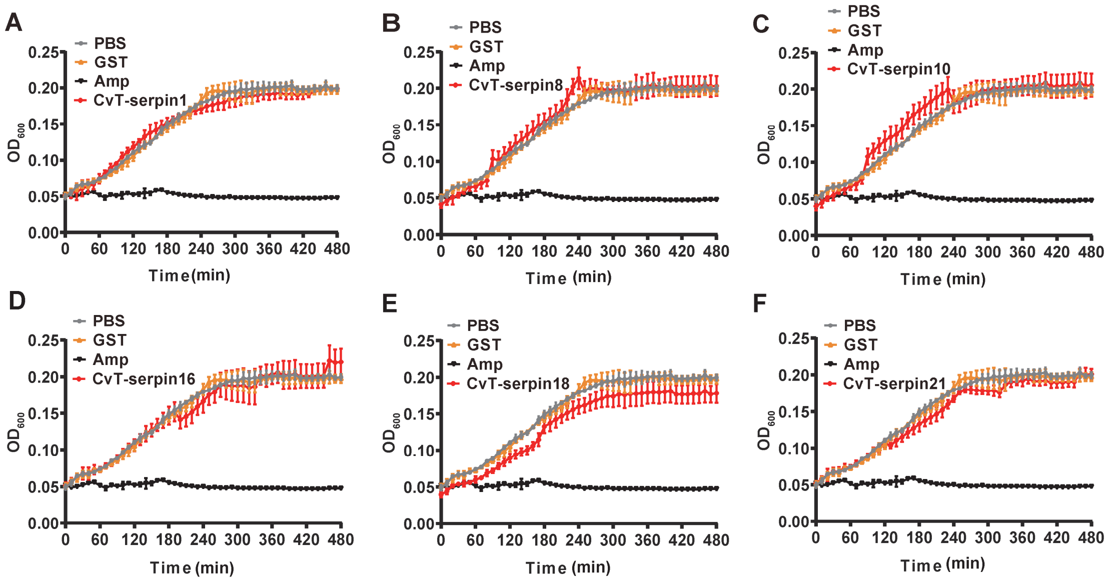

Figure S8. The growth curves of *Escherichia coli* exposed to rCvT-serpin1 (A), rCvT-serpin8 (B), rCvT-serpin10 (C), rCvT-serpin16 (D), rCvT-serpin18 (E), rCvT-serpin21 (F). *E. coli* was cultured to exponential phase, and then diluted with fresh medium to OD600=0.05. 20 µl protein solution contains 15 µg rCvT-serpin1, 30 µg rCvT-serpin8, 10 µg rCvT-serpin10, 10 µg rCvT-serpin16, 30 µg rCvT-serpin18 or 10 µg rCvT-serpin21 was added into 80 µl bacterial suspension. The mixtures were incubated at 37 °C and the absorbance at 600 nm was recorded every 10 min. PBS, GST, and 1 mg ml^{-1} of ampicillin was used as the blank, negative and positive controls, respectively. Error bars represent the mean ± S.D. (N = 3).

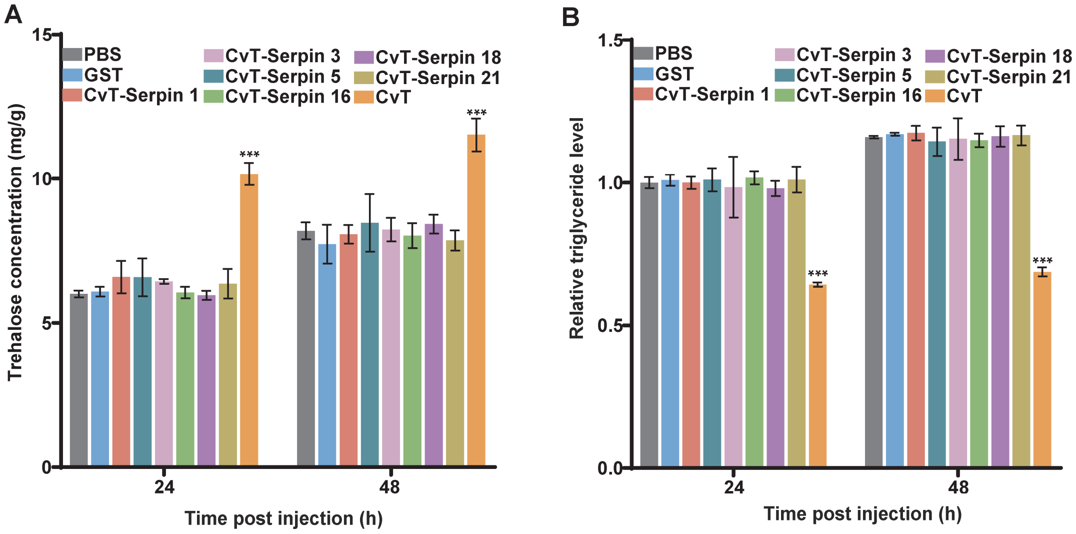

Figure S9. rCvT-serpin1, rCvT-serpin3, rCvT-serpin5, rCvT-serpin16, rCvT-serpin18, rCvT-serpin21 had no effects on trehalose and triglyceride levels of *P. xylostella.* (A) The effect of CvT-serpins and teratocytes' content (CvTC) on trehalose content of *P. xylostella*. (B) The effect of CvT-serpins and CvTC on triglyceride content of *P. xylostella*. All data represent the average of six biological replicates and data are the means ± S.D. Statistical analysis was done by Tukey's test. (*: $P < 0.05$; **: $P < 0.01$; *** $P < 0.001$).
