# Supplementary material for: Parasitoid Serpins Evolve Novel Functions to Manipulate Host Homeostasis
Source: Mol Biol Evol. 2023 Dec 7;40(12):msad269. doi: 10.1093/molbev/msad269 (PMC10735303; doi:10.1093/molbev/msad269)
Supplement: msad269_Supplementary_Data [file msad269_supplementary_data.zip › Supplemental_table_MBE-23-0474 wzwR1.docx]

**Supplementary table S1.** Sites under positive selection in the Reaction Center Loop (RCL) of 19 duplicative *C. vestalis* serpin genes. Sites under positive selection were estimated by the site model in CodeML. The short conserved blocks of the RCL were chosen to minimize the problem of gappy alignment and maximized the sensitivity in detecting valid hits by restricting attention to the most conserved protein regions. The alignment included 37 sites for the RCL of 19 serpin genes, with the amino-acid (Amino-acid) referred to the sequence of Cv-serpin3. Significantly positive selection sites (*P* >0.99) are marked in orange. The dN/dS value (ω) and standard error (S. E.) of the site were calculated for each site.

| Site | Amino-acid | dN/dS | S. E. | Postmean (P) |
| --- | --- | --- | --- | --- |
| 1 | E | 0.05 | 0 | 0.029 |
| 2 | E | 0.115 | 0.056 | 0.113 |
| 3 | G | 0.05 | 0.002 | 0.03 |
| 4 | S | 0.057 | 0.025 | 0.097 |
| 5 | E | 0.05 | 0.001 | 0.029 |
| 6 | A | 0.05 | 0.004 | 0.032 |
| 7 | A | 0.064 | 0.036 | 0.102 |
| 8 | A | 0.051 | 0.008 | 0.049 |
| 9 | A | 0.104 | 0.064 | 0.112 |
| 10 | T | 0.05 | 0.003 | 0.03 |
| 11 | V | 0.142 | 0.085 | 0.129 |
| 12 | V | 0.259 | 0.082 | 0.246 |
| 13 | Q | 2.808 | 3.172 | 0.98 |
| 14 | I | 0.292 | 0.112 | 0.249 |
| 15 | R | 2.496 | 3.031 | 0.983 |
| 16 | L | 0.688 | 1.189 | 0.554 |
| 17 | K | 0.268 | 0.108 | 0.244 |
| 18 | - | 0.991 | 2.08 | 0.394 |
| 19 | - | 0.198 | 0.315 | 0.175 |
| 20 | - | 0.87 | 2.03 | 0.345 |
| 21 | - | 1.514 | 2.423 | 0.749 |
| 22 | - | 2.34 | 3.014 | 0.856 |
| 23 | R | 2.276 | 2.967 | 0.872 |
| 24 | M | 4.415 | 3.393 | 0.998 |
| 25 | A | 1.739 | 2.534 | 0.956 |
| 26 | V | 0.941 | 1.495 | 0.87 |
| 27 | Q | 1.694 | 2.539 | 0.842 |
| 28 | T | 0.212 | 0.174 | 0.211 |
| 29 | E | 2.485 | 3.039 | 0.939 |
| 30 | E | 0.789 | 1.567 | 0.437 |
| 31 | F | 0.077 | 0.048 | 0.107 |
| 32 | I | 0.366 | 0.679 | 0.268 |
| 33 | V | 0.052 | 0.013 | 0.054 |
| 34 | D | 0.05 | 0.007 | 0.041 |
| 35 | R | 0.051 | 0.009 | 0.049 |
| 36 | P | 0.05 | 0.001 | 0.029 |
| 37 | F | 0.05 | 0.003 | 0.031 |

**Supplementary table S2.** Sites under positive selection in the Reaction Center Loop (RCL) of 19 duplicative *C. vestalis* serpin genes according to FEL method. Sites under positive selection were estimated by the FEL method (<https://www.datamonkey.org/>) (Kosakovsky Pond and Frost 2005). A subset of branches containing the Cv-serpin3 were analyzed independently to estimate the synonymous substitution rate (dS) at a site (alpha) and the non-synonymous substitution rate (dN) at a site (beta), with the amino-acid (Amino-acid) referred to the sequence of Cv-serpin3. Significantly positive selection sites (*P* >0.99) are marked in orange. dN/dS MLE, p-value and p-asmp were calculated. dN/dS MLE, the best value for dN/dS that estimated by maximum likelihood estimation; p-asmp. empirical p-value.

| Site | Amino-acid | alpha | beta | alpha=beta | p-value | dN/dS MLE | p-asmp |
| --- | --- | --- | --- | --- | --- | --- | --- |
| 1 | E | 0.92 | 0.004 | 0.007 | 0.05 | 0.004 | 0.0146 |
| 2 | E | 4707.566 | 0.032 | 22.865 | 0.05 | 0 | 0 |
| 3 | G | 470.537 | 0.01 | 22.92 | 0.05 | 0 | 0 |
| 4 | S | 0.054 | 0.031 | 0.034 | 0.6 | 0.583 | 0.5662 |
| 5 | E | 0.021 | 0.012 | 0.013 | 0.6 | 0.588 | 0.6741 |
| 6 | A | 0.966 | 0.01 | 0.027 | 0.05 | 0.011 | 0.0026 |
| 7 | A | 8757.622 | 0.029 | 22.865 | 0.05 | 0 | 0 |
| 8 | A | 688.49 | 0.024 | 23.29 | 0.05 | 0 | 0 |
| 9 | A | 17.151 | 0.052 | 0.057 | 1 | 0.003 | 1 |
| 10 | T | 0.676 | 0.012 | 0.027 | 0.05 | 0.017 | 0.0003 |
| 11 | V | 1.192 | 0.052 | 0.086 | 0.2 | 0.044 | 0.014 |
| 12 | V | 4406.802 | 0.052 | 22.865 | 0.05 | 0 | 0 |
| 13 | Q | 261.986 | 0.174 | 17.742 | 0.55 | 0.001 | 0.4971 |
| 14 | I | 0.143 | 0.049 | 0.059 | 0.5 | 0.34 | 0.3135 |
| 15 | R | 0.152 | 0.101 | 0.113 | 0.6 | 0.66 | 0.6504 |
| 16 | L | 4383.687 | 0.077 | 22.865 | 0.05 | 0 | 0.0053 |
| 17 | K | 0.085 | 0.053 | 0.059 | 0.35 | 0.624 | 0.5205 |
| 18 | - | 805.447 | 0.028 | 22.865 | 0.05 | 0 | 0.0851 |
| 19 | - | 10000 | 0.069 | 147.317 | 0.05 | 0 | 0.0151 |
| 20 | - | 1 | 5 | 3.336 | 0.9 | 5 | 1 |
| 21 | - | 221.293 | 0.149 | 55.435 | 0.5 | 0.001 | 0.5035 |
| 22 | - | 172.827 | 0.091 | 15.491 | 0.25 | 0.001 | 0.3344 |
| 23 | R | 800.21 | 0.144 | 10000 | 0.05 | 0 | 0.4121 |
| 24 | M | 1.353 | 2.75 | 6.307 | 0.04 | 2.033 | 0.9999 |
| 25 | A | 380.537 | 0.081 | 22.967 | 0.05 | 0 | 0.0358 |
| 26 | V | 255.583 | 0.097 | 17.614 | 0.05 | 0 | 0.1129 |
| 27 | Q | 0.112 | 0.141 | 0.128 | 0.85 | 1.261 | 0.8941 |
| 28 | T | 3401.754 | 0.044 | 22.865 | 0.05 | 0 | 0 |
| 29 | E | 0.101 | 0.129 | 0.121 | 0.75 | 1.279 | 0.826 |
| 30 | E | 0.134 | 0.139 | 0.138 | 0.85 | 1.039 | 0.9759 |
| 31 | F | 0.235 | 0.017 | 0.035 | 0.2 | 0.074 | 0.0001 |
| 32 | I | 2607.781 | 0.075 | 22.865 | 0.05 | 0 | 0.0086 |
| 33 | V | 16.578 | 0.016 | 0.041 | 0.05 | 0.001 | 0.0002 |
| 34 | D | 123.862 | 0.013 | 13.495 | 0.05 | 0 | 0 |
| 35 | R | 1.739 | 0.011 | 0.036 | 0.05 | 0.006 | 0 |
| 36 | P | 0.075 | 0.004 | 0.015 | 0.05 | 0.053 | 0.0002 |
| 37 | F | 778.487 | 0.008 | 22.865 | 0.05 | 0 | 0 |

**Supplementary table S3.** List of the species and corresponding resources used for phylogenetic tree. Sequences were retrieved from the NCBI (<https://www.ncbi.nlm.nih.gov/)> and insectbace2.0 (<http://v2.insect-genome.com/>) (Mei et al. 2022).

| Species | Resource | Database | Reference |
| --- | --- | --- | --- |
| *Cotesia vestalis* | GCA_001675545.1 | NCBI | (Shi et al. 2019) |
| *Nasonia vitripennis* | GCA_009193385.2 | NCBI | (Dalla Benetta et al. 2020) |
| *Apis mellifera* | GCA_003254395.2 | NCBI | (Wallberg et al. 2019) |
| *Orussus abietinus* | GCA_000612105.2 | NCBI | (Misof et al. 2014) |
| *Monomorium pharaonis* | GCA_013373865.1 | NCBI | (Gao et al. 2020) |
| *Tribolium castaneum* | GCA_000002335.3 | NCBI | (Kim et al. 2010) |
| *Bombyx mori* | GCA_027497115.1 | NCBI | (Lu et al. 2020) |
| *Drosophila melanogaster* | GCA_000001215.4 | NCBI | (Adams et al. 2000) |
| *Homo sapiens* | GCA_000001405.29 | NCBI | (Nurk et al. 2022) |

**Supplementary table S4.** Table list of primers used in this research, the GenBank accession number of the corresponding gene was listed.

| **Primer name** | GenBank accession | **Primer sequence (5’-3’)** | **Purpose** |
| --- | --- | --- | --- |
| CvT-serpin1-F | OQ594896 | ATGGCAAAATCACGAGGA | Cloning |
| CvT-serpin1-R | / | TTAATTTGTTTCAGCAGGT | Cloning |
| CvT-serpin3-F | OQ594898 | ATGGCTGAAAAACCTGACTATC | Cloning |
| CvT-serpin3-R | / | TTATAGCTCGTCTTTGTTGGT | Cloning |
| CvT-serpin5-F | OQ594900 | GCCAATACTATAGCACTGTATAGGAA | Cloning |
| CvT-serpin5-R | / | TTATGTTTCTATGGGATTATACAC | Cloning |
| CvT-serpin8-F | OQ594903 | ATGATAAAAAAACTTCTACTCAGTGG | Cloning |
| CvT-serpin8-R | / | TTAATTGGAAGGATCAGTTACGCG | Cloning |
| CvT-serpin10-F | OQ594905 | ATGATGATAAAGTTGATATTAATCGC | Cloning |
| CvT-serpin10-R | / | TCAAACTGCCGTAGGGTCCATTAC | Cloning |
| CvT-serpin16-F | OQ594911 | ATGAATCGAAATAATTGGACTT | Cloning |
| CvT-serpin16-R | / | TTACTTAAACATATTGGTAAAAAGTGG | Cloning |
| CvT-serpin18-F | OQ594913 | ATGAACTTCAAAGCATGGTTTATT | Cloning |
| CvT-serpin18-R | / | TCATTTTGATGGATCAACGACGTGT | Cloning |
| CvT-serpin21-F | OQ594916 | ATGAAACTCCATTTAATTGCATTG | Cloning |
| CvT-serpin21-R | / | TTAATAATACATCGTCCCAGGATT | Cloning |
| CvT-serpin1-qPCR-F | / | ACCAACAGCGAATCCCAATAC | qPCR |
| CvT-serpin1-qPCR-R | / | TAGGGCATAAGACCGCTGG | qPCR |
| CvT-serpin3-qPCR-F | / | ATTGGGTTTTGGAGAATACAAAT | qPCR |
| CvT-serpin3-qPCR-R | / | TCTCCATGAACAAAGTGGGCT | qPCR |
| CvT-serpin5-qPCR-F | / | GCGGCCTGAATTCAAAGAAT | qPCR |
| CvT-serpin5-qPCR-R | / | TTCGATTGAATTGGCGTTT | qPCR |
| CvT-serpin8-qPCR-F | / | GTGGCATGATAGCGGCTT | qPCR |
| CvT-serpin8-qPCR-R | / | TTCAGTGTTTCCTCGCGC | qPCR |
| CvT-serpin10-qPCR-F | / | CGGCGATGAAAATGACGAT | qPCR |
| CvT-serpin10-qPCR-R | / | TTTGTGAAATCAGCCGTGTT | qPCR |
| CvT-serpin16-qPCR-F | / | CAATGGCCTTTGGGCATC | qPCR |
| CvT-serpin16-qPCR-R | / | TTCAACTCTTTTCAAACCGC | qPCR |
| CvT-serpin18-qPCR-F | / | AGCTTGGGCAAAAGGGTT | qPCR |
| CvT-serpin18-qPCR-R | / | GTTTCTGGTGCTTCCTTTGC | qPCR |
| CvT-serpin21-qPCR-F | / | TTCAAAGCGATGTGGGTCC | qPCR |
| CvT-serpin21-qPCR-R | / | GATCTTGCAAACCATCTTTCC | qPCR |
| Cv-18srRNA-qPCR-F | JX399880.1 | ATGTCTGCCTTATCAACTGTCG | qPCR |
| Cv-18srRNA-qPCR-R | / | TCCTTGGATGTGGTAGCCG | qPCR |
| Cv-tubulin-qPCR-F | MT459787 | CCTACTGGTCAAATGTTCCG | qPCR |
| Cv-tubulin-qPCR-R | / | ATCACAGCCCTCAGACTCTCG | qPCR |
| 16S rRNA-F | / | GTGCCAGCMGCCGCGGTAA | qPCR |
| 16S rRNA-R | / | CCGTCAATTCMTTTGAGTTT | qPCR |
| PxHK-qPCR-F | XM_048629578.1 | CAATAGCCAGACGAGGGGACG | qPCR |
| PxHK-qPCR-R | / | ATGCCCCCCATTCTGTGTTT | qPCR |
| PxTPS-qPCR-F | XM_048624501.1 | TAACTGGATTAGACAGACCGCT | qPCR |
| PxTPS-qPCR-R | / | CAGGCAGTAGTCTGTGATGTGG | qPCR |
| PxTRET- qPCR-F | XM_011555991.3 | ACAGGGGAAACGGAAGTAGTAT | qPCR |
| PxTRET- qPCR-R | / | TATCAAAAACTCATTTATTTCT | qPCR |
| PxNAGA-qPCR-F | XM_011562037.1 | ACCCCGATGAGTGTATCAGTGAG | qPCR |
| PxNAGA-qPCR-R | / | CCCCACAGCCTTCATTCC | qPCR |
| PxN-Smase-qPCR-F | XM_011570071.1 | GTCCTGGGTTCTGGTCTGTG | qPCR |
| PxN-Smase-qPCR-R | / | CATATTTGATGCGGCAGAGAC | qPCR |
| PxPSD-qPCR-F | XM_011565434.1 | ACAAAGGCTGCGTCCACAC | qPCR |
| PxPSD-qPCR-R | / | CCAGTTTCCCCCAAATCCT | qPCR |
| Px-β-actin-F | NM_001309101 | TGGCACCACACCTTCTAC | qPCR |
| Px-β-actin-R | / | CATGATCTGGGTCATCTTCT | qPCR |
| Px-β-tublin-F | EU127912 | GACGCATGTCCATGAAGGAG | qPCR |
| Px-β-tublin-R | / | CCAATGCAAGAAAGCCTTGC | qPCR |
| CvT-serpin1-BamHI | / | CAGGGGCCCCTGGGATCCATGGCAAAATCACGAGTGAAT | Vector construction |
| CvT-serpin1-Xhol | / | ACGATGCGGCCGCTCGAGTTAATTTGTTTCAGCAGGTGAAGT | Vector construction |
| CvT-serpin3-BamHI | / | CAGGGGCCCCTGGGATCCATGAGACTAAATTTATCAAGT | Vector construction |
| CvT-serpin3-Xhol | / | ACGATGCGGCCGCTCGAGTTAAATTATCTCTTTAACATGA | Vector construction |
| CvT-serpin5-BamHI | / | CAGGGGCCCCTGGGATCCATGCTTTATTTTGGAGCAAGAG | Vector construction |
| CvT-serpin5-Xhol | / | ACGATGCGGCCGCTCGAGTTATGTTTCTATGGGATTATACACTTG | Vector construction |
| CvT-serpin8-BamHI | / | CAGGGGCCCCTGGGATCCATGACGCTTGAAGTTAAATGTGAG | Vector construction |
| CvT-serpin8-Xhol | / | ACGATGCGGCCGCTCGAGTTAATTGGAAGGATCAGTTACGCG | Vector construction |
| CvT-serpin10-BamHI | / | CAGGGGCCCCTGGGATCCATGATGATAAAGTTGATATTAATCGC | Vector construction |
| CvT-serpin10-Xhol | / | ACGATGCGGCCGCTCGAGTCAAACTGCCGTAGGGTCCATTAC | Vector construction |
| CvT-serpin16-BamHI | / | CAGGGGCCCCTGGGATCCATGAATCGAAATAATTGGACTT | Vector construction |
| CvT-serpin16-Xhol | / | ACGATGCGGCCGCTCGAGTTACTTAAACATATTGGTAAAAAGTG | Vector construction |
| CvT-serpin18-BamHI | / | CAGGGGCCCCTGGGATCCATGAACTTCAAAGCATGGTTTATT | Vector construction |
| CvT-serpin18-Xhol | / | ACGATGCGGCCGCTCGAGTCATTTTGATGGATCAACGACGTGT | Vector construction |
| CvT-serpin21-BamHI | / | CAGGGGCCCCTGGGATCCATGAAACTCCATTTAATTGCATTG | Vector construction |
| CvT-serpin21-Xhol | / | ACGATGCGGCCGCTCGAGTTAATAATACATCGTCCCAGGATT | Vector construction |

**Supplementary table S5.** Table list of selected specific amino acid sequence of each CvT-serpin used as antigen to produce rabbit polyclonal antibodies

| **Gene name** | **Antigenic region** | **Amino acid sequence** |
| --- | --- | --- |
| CvT-Serpin1 | 281-297 aa | ARRGFRQAPAAGETVGI |
| CvT-Serpin3 | 3-20 aa | EKPDYHTFTSNLFKAVTE |
| CvT-Serpin5 | 80-94 aa | TNNQITEIIEPDDLE |
| CvT-Serpin8 | 209-223 aa | GKFKFGYIAHPKAEY |
| CvT-Serpin10 | 5-18 aa | IASKSIKYPQRFVS |
| CvT-Serpin16 | 415-429 aa | STKRYVEPVKPGFTS |
| CvT-Serpin18 | 2-17 aa | KAVSRRKKNGLYQTTKG |
| CvT-Serpin21 | 331-342 aa | NEEGSEASTTSA |

The antigenic region was selected after removing the signal peptides of each CvT-serpin.

**References**

Adams MD, Celniker SE, Holt RA, Evans CA, Gocayne JD, Amanatides PG, Scherer SE, Li PW, Hoskins RA, Galle RF, et al. 2000. The genome sequence of *Drosophila* *melanogaster*. *Science* **287:**2185–2195.

Dalla Benetta E, Antoshechkin I, Yang T, Nguyen HQM, Ferree PM, Akbari OS. 2020. Genome elimination mediated by gene expression from a selfish chromosome. *Sci. Adv.* **6:**eaaz9808.

Gao Q, Xiong Z, Larsen RS, Zhou L, Zhao J, Ding G, Zhao R, Liu C, Ran H, Zhang G. 2020. High-quality chromosome-level genome assembly and full-length transcriptome analysis of the pharaoh ant *Monomorium pharaonis.* *GigaScience* **9:**giaa143.

Khan S, Sowpati DT, Srinivasan A, Soujanya M, Mishra RK. 2020. Long-read genome sequencing and assembly of *Leptopilina boulardi:* A specialist *Drosophila* parasitoid. *G3 Bethesda Md* **10:**1485–1494.

Kim HS, Murphy T, Xia J, Caragea D, Park Y, Beeman RW, Lorenzen MD, Butcher S, Manak JR, Brown SJ. 2010. BeetleBase in 2010: Revisions to provide comprehensive genomic information for *Tribolium castaneum*. *Nucleic Acids Res.* **38:**D437-442.

Lu F, Wei Z, Luo Y, Guo H, Zhang G, Xia Q, Wang Y. 2020. SilkDB 3.0: visualizing and exploring multiple levels of data for silkworm. *Nucleic Acids Res.* **48:**D749–D755.

Kosakovsky Pond SL, Frost SDW. 2005. Not so different after All: A comparison of methods for detecting amino acid sites under selection. *Mol. Biol. Evol.* **22:**1208–1222.

Mei Y, Jing D, Tang S, Chen X, Chen H, Duanmu H, Cong Y, Chen M, Ye X, Zhou H, et al. 2022. InsectBase 2.0: A comprehensive gene resource for insects. *Nucleic Acids Res.* **50:**D1040–D1045.

Misof B, Liu S, Meusemann K, Peters RS, Donath A, Mayer C, Frandsen PB, Ware J, Flouri T, Beutel RG, et al. 2014. Phylogenomics resolves the timing and pattern of insect evolution. *Science* **346:**763–767.

Nurk S, Koren S, Rhie A, Rautiainen M, Bzikadze AV, Mikheenko A, Vollger MR, Altemose N, Uralsky L, Gershman A, et al. 2022. The complete sequence of a human genome. *Science* **376:**44–53.

Shi M, Wang Z, Ye X, Xie H, Li F, Hu X, Wang Z, Yin C, Zhou Y, Gu Q, et al. 2019. The genomes of two parasitic wasps that parasitize the diamondback moth. *BMC Genomics* **20:**893.

Wallberg A, Bunikis I, Pettersson OV, Mosbech M-B, Childers AK, Evans JD, Mikheyev AS, Robertson HM, Robinson GE, Webster MT. 2019. A hybrid de novo genome assembly of the honeybee, *Apis mellifera*, with chromosome-length scaffolds. *BMC Genomics* **20:**275.
